# An Atacama subsurface tephra layer reveals how life colonized Kenorland in the Neoarchean

**DOI:** 10.1038/s41598-025-24288-x

**Published:** 2025-12-09

**Authors:** Armando Azua-Bustos, Carlos González-Silva, Daniel Carrizo, Laura Sánchez-García, Maite Fernández-Sampedro, Thanh Quy Dang, Cristian Vargas-Carrera, Victoria Muñoz-Iglesias, María Paz Martin-Redondo, Pedro Mustieles-del-Ser, Olga Prieto-Ballesteros, Jacek Wierzchos

**Affiliations:** 1 Centro de Astrobiología (CAB), CSIC-INTA, Carretera de Ajalvir km4, Torrejón de Ardoz, Madrid, 28850 Spain; 2 Facultad de Ciencias, Universidad de Tarapacá, Arica, Chile; 3 Consultora ProBiota E.I.R.L., Iquique, Chile; 4 Laboratoire de Planétologie et Géosciences, CNRS, LPG UMR 6112, Nantes Université, Univ Angers, Le Mans Université, Nantes, 44000 France; 5 Museo Nacional de Ciencias Naturales (CSIC), Madrid, 28006 Spain; 6 Centro de Astrobiología (CSIC-INTA), Madrid, Spain

**Keywords:** Ecology, Microbiology

## Abstract

Much has been published on the microbiology of the Atacama Desert, the driest and oldest place on Earth, but little is known about how microbial life is able to permanently colonize this extremely harsh environment. Given that wind transports vast amounts of viable microbial cells from the Pacific Ocean across the entire Atacama on a daily basis, it is difficult to identify the true native microbial inhabitants from recent arrivals. Here, we present a unique site—Mancha Blanca—located at the western edge of the Coastal Range of this desert. Containing a Miocene tephra subsurface layer, this site’s unique differential habitability unveils the fate of new arrivals, and thus, the identity and metabolic strategies of the species that are eventually able to adapt and become some of the true inhabitants of the Atacama. Our findings show that the extreme conditions of the Atacama stochastically select for species arriving from the Pacific Ocean that already possess the molecular mechanisms needed to tolerate salinity, and thus are capable of inhabiting the driest desert on Earth. The colonization processes still detectable in the regions studied allow us to suggest the mechanistic and evolutionary pathways that may have been involved in the colonization of Kenorland by microbial life from the Panthalassic Ocean during the Neoarchaean. We propose the Coastal Range/hyperarid core of the Atacama as a model system for understanding the colonization of land 2.6 billion years ago.

**Supplementary Information:**

The online version contains supplementary material available at 10.1038/s41598-025-24288-x.

## Introduction

 Microbial life was present on land during the Proterozoic eon^1^, with well documented microfossils representative of terrestrial ecosystems 2.6 billion of years ago^2^. Since microbial life flourished in the oceans since at least 3.8 billion years^3^, the mechanistic and evolutionary processes that supported life in the critical step to adapt from an environment with an ample availability of water (the Panthalassic Ocean) to a much drier environment (Kenorland) are yet to be understood. Our first observations on how this process may have taken place started with the discovery of unusual microorganisms in the Coastal Range of the Atacama Desert, the driest and oldest desert on Earth^4^: (a) *Cyanidium* sp. Atacama, an ancient microalgae thought to still be in the process of secondary endosymbiosis that gave rise to modern chloroplasts^5^, (b) *Dunaliella atacamensis*, the only known subaerial member of the *Dunaliella* genus, with clear morphological and physiological adaptations to survive outside the water^6^ and (c) *Gloecapsopsis dulcis*, a hypolithic cyanobacteria extremely resistant to desiccation^7–12^ with clear molecular evidences of a marine origin^13^.

These findings first allowed to propose exaptation (features that now enhance fitness but were not built by natural selection for their current role^14^ as the evolutionary mechanism allowing these species to colonize the coasts of the driest and oldest desert on Earth^13^. We later reported that wind-transported dust was the mechanism that allowed the colonization of the Atacama by microorganisms coming from the Pacific Ocean^15^ in this region, coherent with a later finding of a number of biosignatures, DNA sequences and microbial isolates that clearly showed the colonization of the beachfront by microbial species coming from the Pacific^16^. Here we report the findings of Mancha Blanca, a site located in the Coastal Range/hyperarid core transition of the Atacama, that show the fate of the microbial species that arrive from the Pacific Ocean, in which the extreme conditions of the Atacama stochastically select for those species that already had the molecular mechanisms that allowed them to adapt, and then colonize the land.

## Results and discussion

**Fig. 1:**
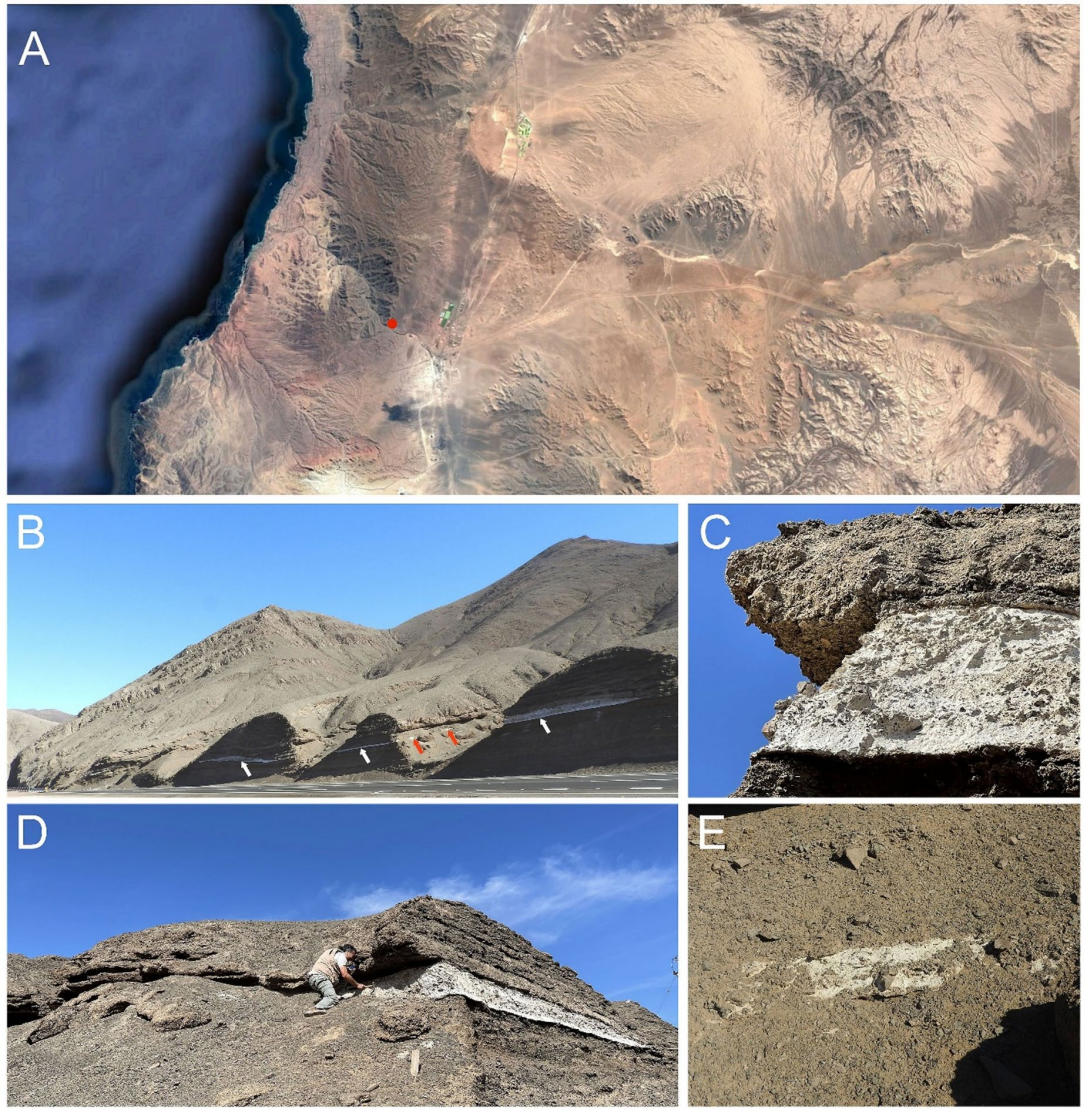
Location and visual aspect of Mancha Blanca Site. **A**, Satellite map of the studied region. The red dot show the location of Mancha Blanca. **B**. panoramic view of the tephra layer, showing the artificially exposed (AE) (white arrows) and the naturally exposed (NE) portions (red arrows). **C**, detailed view of one of the AE portions. **D**, detailed view of one of the AE and NE portions. **E**, detailed view of another of the NE portions. The satellite image shown in Panel A was obtained from Google Earth Pro, Version 7.3.6.

Mancha Blanca is located at Quebrada La Negra (Fig. 1A and B), a ravine that cuts through the eastern hills of the Coastal Range in the Atacama Desert, approximately 11 km from the Pacific Ocean. It lies in the transitional zone between the Coastal Range and the hyperarid core of the Atacama. This site is unique because it contains a white-colored lenticular layer (Fig. 1B, C, and D), visible in slope outcrops where it is intercalated between ancient Miocene alluvial gravels and sand sediments, which are cut by Quaternary gullies. Notably, this layer was recently exposed artificially (Fig. 1B) during the reconstruction of a road connecting the coast to the hyperarid core of the desert in 2012^17^. Similar deposits in this region are disposed in angular discordance over the Mesozoic stratified volcanic rocks of the La Negra Formation, which, in turn, discordantly underlie the Quaternary sediments in the sampling area (Fig. S1). These deposits are composed of poorly sorted sediments, partially cemented by chlorides, sulfates, and occasionally carbonates^18^. They originate from ancient colluvial cones or alluvial fans whose runoffs generally drain toward the axis of the Salar del Carmen depression (N–S). The age of these deposits has been precisely determined from biotites within white volcanic ash strata, corresponding to the Pliocene period^19,20^. Similar tephra, containing variable amounts of vitroclasts (shards)^21^, occur in tabular forms or as elongated, irregular levels, and sometimes display palaeo-channels and other features indicative of reworking of the original fall deposits^21^.

**Fig. 2:**
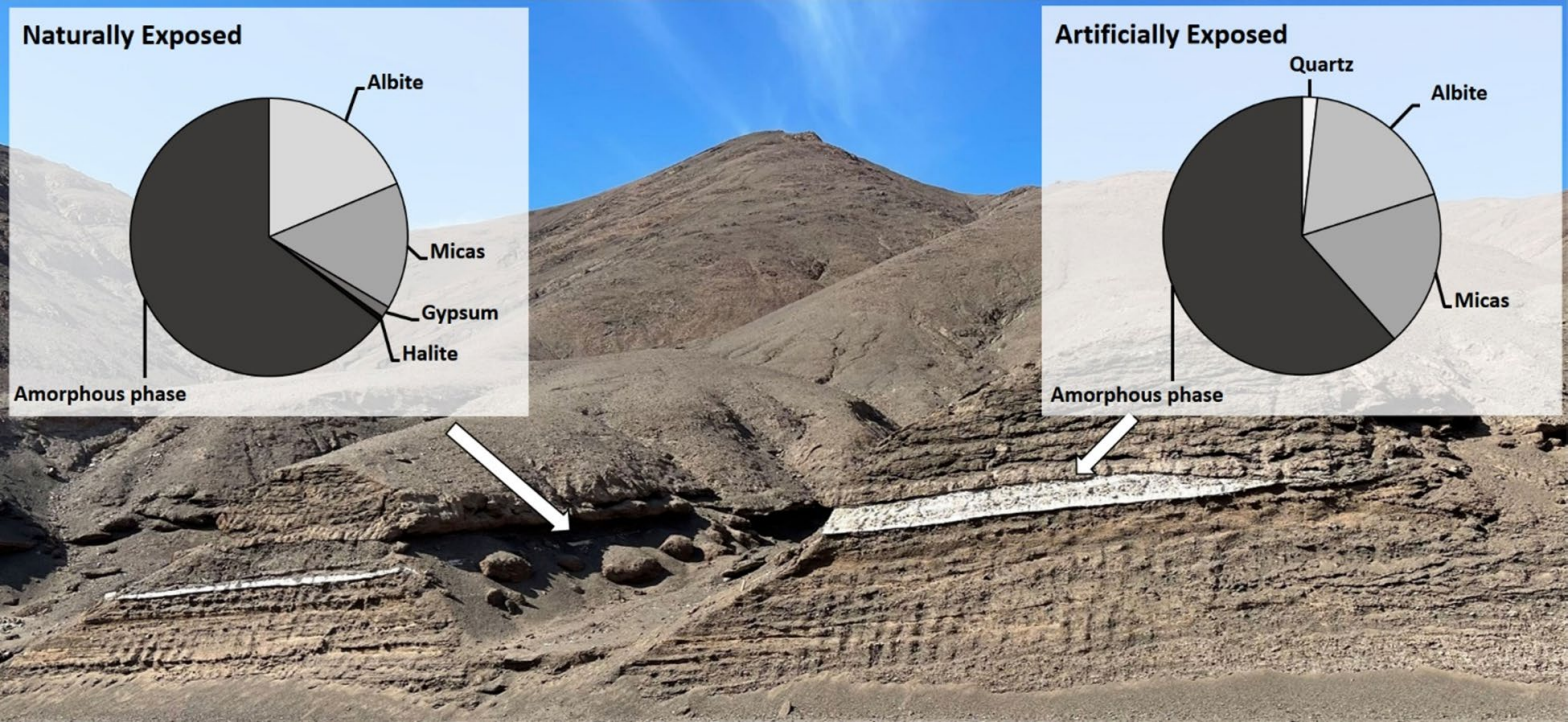
Mineralogical composition of Mancha Blanca tephra layer. Minerals were determined by XRD and Raman spectroscopy and X-ray fluorescence, as detailed in figure S2, S3 and Table S1.

 Important for our analyses, small sections of the tephra layer remain naturally exposed to the environment in the two gullies that cross this site, as they were before the road was built in this sector (red arrows in Fig. 1B, detailed in Fig. 1E), thus providing two distinct sampling points: artificially exposed (AE) and naturally exposed (NE). X-Ray diffraction (Fig S2), Raman spectroscopy (Fig S3) and X-Ray fluorescence (Table S1) analyses showed that the main minerals present in both the AE and NE samples are albite, micas (mostly sericite derived from muscovite), and amorphous silica, with small amounts of gypsum and halite present in the NE samples, and quartz in the AE samples (Fig. 2).

Automatic data-loggers embedded in the tephra horizon both in the AE and NE areas showed the typical environmental daily variations of this region (Fig. S4A to S4B). AE and NE temperatures were similar (Fig. S4C-S4E), and rather low compared with other sites of the Atacama, in which soil surface temperatures can easily reach 50°C^22^. Relative humidity raised up to 98% (a_w_ 0.98) during the night and early hours of the morning (Fig. S4A), values which have shown to be enough to sustain microbial activity in this region^7^. A significant difference was detected in the amount of water available for microbial life in favor of the AE tephra (Fig. S4F-S4H).

**Fig. 3:**
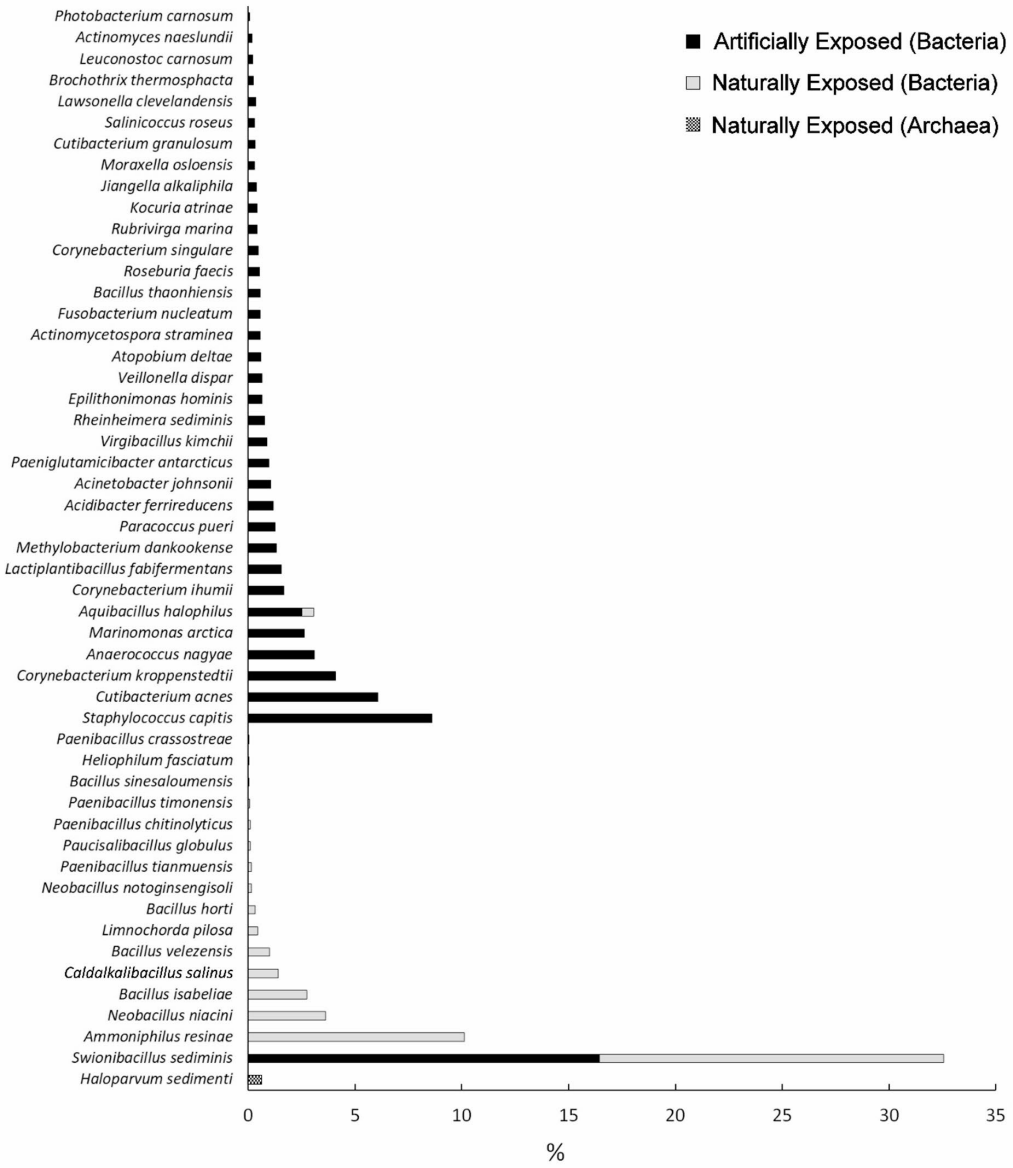
Percentage of OTU’s found in Mancha Blanca Tephra samples. Bars show the percentage of the total bacterial and archaeal sequences found by NGS, and the ID of the most closely related species in each case.

Culture independent Next Generation Sequencing (NGS) analyses unveiled the presence of mostly heterotrophic bacteria in AE and NE samples. Only a single OTU (Operational Taxonomic Unit), closely related to the archaea *Haloparvum sedimenti*, an extremely halophilic and thermophilic aerobic species first reported in halites of a salt mine in China^23^, was present in the NE samples as representative of this domain of life (Fig. 3). No eukaryotes or cyanobacteria were found using this technique in any of the samples analyzed.

NGS results first unveiled the high disparity of microbial OTU’s found in the AE versus NE samples, with only two out of fifty bacterial OTU’s (two sequences closely related to *Swionibacillus sediminis* and *Aquibacillus halophilus*) shared between the two exposure conditions (Fig. 3). The OTU closest to *Swionibacillus sediminis* (a non-motile, strictly aerobic bacterium isolated from marine sediments of the south-west Indian Ocean^24^, was also the predominant OTU in both sample types, with *Aquibacillus halophilus* reported as a strictly aerobic moderately halophilic bacterium isolated from a hypersaline lake in Iran^25^.

As for the remaining bacterial relatives of the OTU`s found in the NE samples, *Bacillus isabeliae*, *Bacillus sinesaloumensis* and *Neobacillus notoginsengsoli* are halophilic soil species^26–28^, while *Bacillus velezensis*,* Limnochorda pilosa*, *Bacillus horti* and *Paucisalibacillus globulus* are halotolerant and alkalitolerant/alkaliphilic bacteria isolated from soil or brackish environments^29–32^. In turn, *Ammoniphilus resinae*, *Neobacillus niacini*, *Caldalkalibacillus salinus*, *Paenibacillus tianmuensis*, *Paenibacillus tianmuensis*,* Paenibacillus timonensis* and *Heliophilum fasciatum* are bacteria isolated from different soils, water logged or lake sediments around the world^33–40^, with the most taletelling case on the geographical origin of these species been that of *Paenibacillus crassostreae*, an aerobic bacterium isolated from the Pacific *oyster Crassostrea gigas*^41^.

In stark contrast, only two OTU’s of the AE tephra have been reported in soils elsewhere; *Actinomycetospora straminea*, and *Bacillus thaonhiensis*^42,43^, with most of the AE OTU’s reported as part of the human microbiota; OTU’s closely related to bacterial species such as *Cutibacterium acnes*, *Staphylococcus capitis*,* Corynebacterium kroppenstedtii*, *Anaerococcus nagyae*, *Corynebacterium ihumii*, *Methylobacterium dankookense*,* Chryseobacterium hominis*,* Atopobium deltae*,* Fusobacterium nucleatum*,* Roseburia faecis*, *Corynebacterium singulare*, *Moraxella osloensis*, *Cutibacterium granulosum*, *Lawsonella clevelandensis* and *Actinomyces naeslundii*^44–58^ or species found in nature, but also reported as opportunistic human pathogens or in industrial processes, such as *Lactiplantibacillus fabifermentans*, *Paracoccus pueri*, *Acidibacter ferrireducens*, *Virgibacillus kimchi*, *Acinetobacter johnsonii*, *Veillonella dispar*, Kocuria atrinae, *Brochothrix thermosphacta*, *Leuconostoc carnosum*, and *Photobacterium carnosum*^59–68^.

Only six bacterial AE OTU’s are related to species reported in saline, ocean and other environments; *Marinomonas arctica*, *Paeniglutamicibacter terrestris*, *Rheinheimera sediminis*, *Rubrivirga marina*, *Jiangella alkaliphila* and *Salinicoccus roseus*^69–74^, a finding which suggest that these OTUs may be in fact be representatives of the naturally exposed tephra, but still detectable in the artificially exposed areas.

**Fig. 4:**
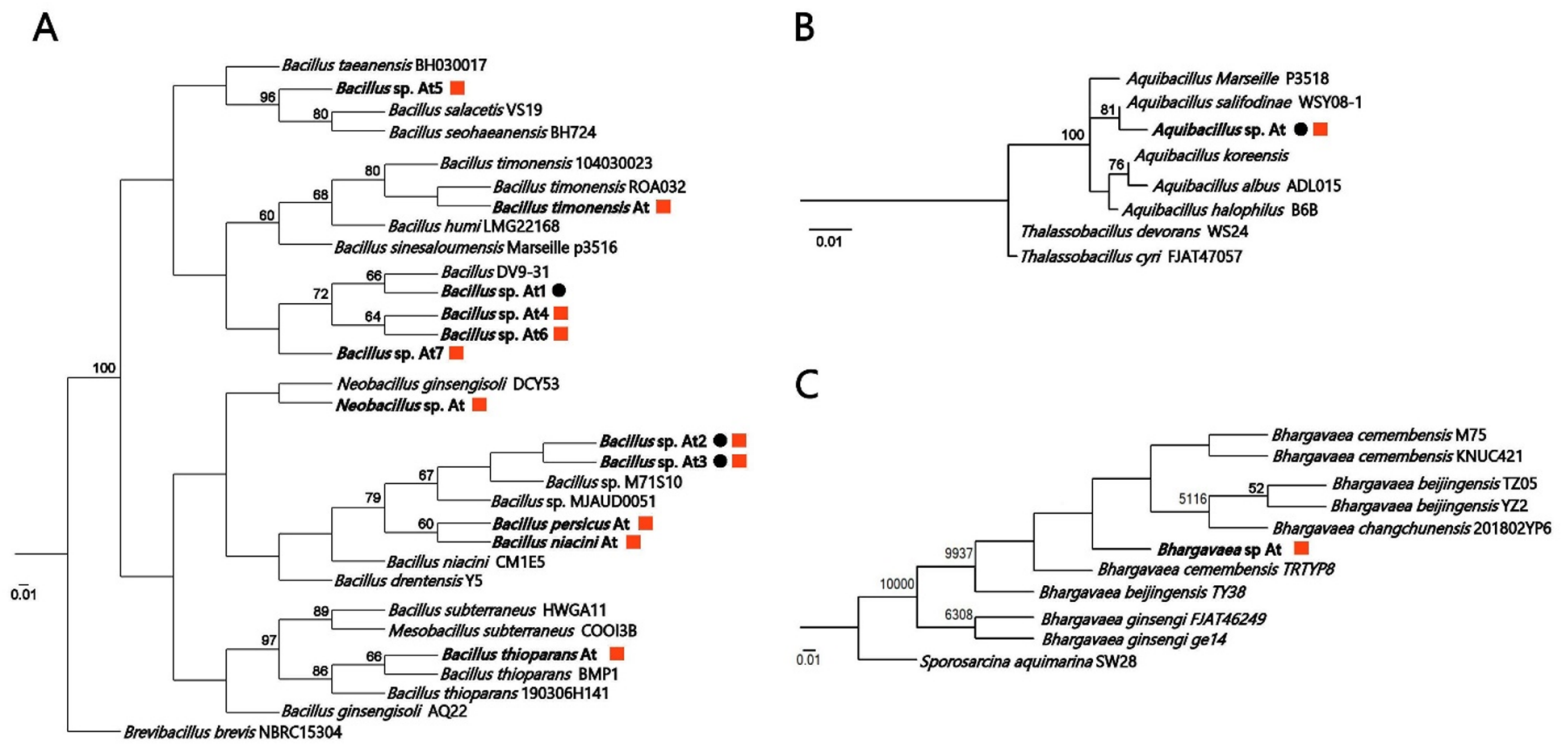
Closest phylogenetic matches of microbial isolates found in Mancha Blanca samples. Phylip neighbor joining phylogenetic trees obtained from the aligned 16 S rRNA gene sequences of Mancha Blanca isolates and related species. Numbers on the nodes represent bootstrap values with 1000 replicates. Bacterial isolates; **(A)** *Bacillus* and *Neobacillus*, **(B)** *Aquibacillus*, and **(C)** *Bhargavaea*. Red squares denote species isolated from naturally exposed samples, while black circles denote species isolated from artificially exposed samples.

Culture of AE and NE tephra samples on different growing media also showed the stark differences in microbial diversity unveiled by NGS sequencing. Of the fourteen isolates obtained, ten were found exclusively in the NE samples, only one exclusively in the AE samples, and three shared among the AE and NE samples (Fig. 4); *Aquibacillus*, (which 16 S rRNA sequence is the same that the corresponding OTU found by NGS) and two species of *Bacillus*, spp. At2 and At3. Taking in account the habitat of *Aquibacillus*, it may also be the case that the three shared species are also representatives of the NE tephra which are also found in the AE samples (in accordance with NGS results), as *Bacillu*s species can easily disperse using spores.

The *Aquibacillus* isolate is of particular interest as a recent pan-genomic analyses of eight *Aquibacillus* strains^75^ showed that all the studied species have the metabolic pathways to synthetize a number of compatible solutes such as betaine, ectoine, glutamate, and proline, known to be involved in the tolerance of saline environments, but also, critically in this case, desiccation tolerance^8,76^. Similarly, the OTUs detected by NGS in the NE samples also have the ability to withstand desiccation due to their ability to tolerate high salinity (i.e., NaCl, KNO_3_, Mg^2+^), as is the case of *Swionibacillus sediminis*, (isolated from marine sediments of the south-west Indian Ocean)^24^, *Bacillus isabeliae*, (an halophilic bacterium isolated from a sea salt evaporation pond)^26^, *Caldalkalibacillus salinus*, (an alkalophilic bacteria isolated from a saline sediment sample collected in China)^36^, *Bacillus velezensis* (capable of growing in NaCl concentrations of up to 12 % w/v)^29^, *Bacillus horti*, (an alkaliphilic bacillus with optimum growth at pH 8 to10 and up to 10% of NaCl)^31^, which also applies to the archaea *Haloparvum sedimenti*, isolated from a salt mine in China, which requires an optimum of 3.4 M of MgCl_2_ and NaCl^23^.

The evident disparity in the microbial diversity of both exposures conditions was also evident when a range of biosignatures was inspected in these samples. The total organic content (TOC % dw) was five times higher in the NE (0.25%) vs. the AE (0.05%) samples (Fig. S5), a second line of evidence consistent with the hypothesis that the microbial species at the NE areas have already adapted, and growing in their current abiotic environment.

 Low amounts of hydrocarbons were found in all samples, with a range of 0.12 to 4.11 µg g^−1^ dw for *n*-alkanes, again with higher concentrations found in the NE samples (Table S2). A similar pattern was found for isoprenoids; in which the higher abundance of this compounds (e.g., pristane, phytane and squalene) in the NE samples, suggested the presence of photoautotrophs (pristane and phytane) and archaea (squalene) (Fig. S6). As pristane and phytane are biosignatures mainly originated from the degradation of phytol from phototrophs^77,78^, their presence in the NE samples is intriguing, as nor microscopy or culture dependent or independent methods detected their presence. This finding, along with the depleted δ^13^C value (range − 25.7 to −26.6‰) of TOC, that are only detectable in the NE samples (Table S3), suggest that pristine and phytane may be the remnants of the phototrophs that inhabited this site before it was covered with subsequent sediments. In the case of squalene, considered to be mainly produced by archaea^79^, (methanogenic, halophilic or thermoacidophilic)^80,81^, its presence is in agreement with the archaeal OTU detected by NGS only in the NE samples.

Hopane series (C_27_ to C_35_) with a mean value of 0.02 µg g^−1^ dw, were also found only in the NE samples (Fig. S7). The major precursors of these compounds are bacteriohopanetetrol found in the lipids membranes of prokaryotic organisms with a similar function than sterols in eukaryotes^82^, with the original biological configuration 17β(H), 21β(H), been unstable during organic matter diagenesis. Thus, the presence of the more thermodynamically stable configuration in these samples, (e.g., 17α(H), 21β(H)) associated with the R and S epimers at C_22_ position (from C_31_ to C_35_), suggest that these molecules are ancient buried hopanes, with a high degree of maturation/diagenesis.

 The acid fraction was more abundant than the hydrocarbons fraction, and composed of linear short chain fatty acids (C_14:0_, C_16:0_ and C_18:0_), terminal branched fatty acids (*iso*/*anteiso*) with C_14:0_ to C_17:0_ chain lengths and unsaturated fatty acids (C_15:1_ to C_19:1_), again with higher values in the NE samples (Fig. S8, Table S4). The higher amount of unsaturated acids in the NE samples suggest the active metabolism of extant cells^83^ (consistent with species well adapted to their environment, also amenable to be grown as aforementioned), while the detection of terminal branched *iso/anteiso* (C_15_) fatty acids in all samples related to the presence of gram positive Firmicutes^84^ (in agreement with the OTU’s closest to *Bacillus*, detected by NGS, and the *Bacillus* isolates found by culturing), again with higher amounts in the NE samples.

 Steroids such as cholesterol (and degradation products, coprostanol) as well as phytosterols (β-sitosterol and stigmastanol) were present in trace amounts (Table S4), and again, only detectable in the NE samples. Functionalized lipids (e.g., fatty acids and alkanols) that are prone to suffer degradation (to non-functionalized molecules) were also present in higher abundance in the NE samples (Table S4 and Table S5), suggesting either a higher biomass or a better preservation over time of these lipids in these sample.

In conclusion, in agreement with the South American continent been located more or less in the same latitude for the last 150 million years^85^, thus affected by similar global climatic conditions, these findings suggest that the microorganisms present in the NE tephra horizon first arrived transported by wind from the Pacific Ocean after (or while) these volcanic ashes were deposited, in a time with no human presence, but before been covered by more recent sediments, having enough time to adapt and colonize its new habitat. This hypothesis agrees with the finding that of most of the species relative to the OTU’s found in the NE tephra have been reported from marine/watery-saline environments, without a single case of species that later arrived in the Anthropocene, as is the case of the AE tephra. In contrast, most of the OTUs found in the AE tephra, which either arrived from the road immediately adjacent to the site, and/or carried by the wind coming from the Pacific Ocean (thus crossing the nearby coastal City of Antofagasta), have arrived in modern times to perish on the exposed tephra, explaining why just remnants of their DNA can be found, without any evidences of active metabolism or cultivable species.

This entire process most certainly happens elsewhere, but given the extremely harsh environment of the Atacama (which in some site is close to sterility^22^, it is still detectable/measurable compared to other environments, in which competition has caused all land niches to be already filled, making the colonization process much harder to observe.

Thus, considering; (a) the high disparity of microbial species found by culture dependent and independent methods between the naturally exposed and the artificially exposed tephra, (b) that the geochemical characteristics between the naturally exposed and the artificially exposed tephra don’t seem different enough to explain its vastly divergent microbial diversity, (c) that the availability of water for microbial life doesn’t favor the naturally exposed areas (in fact, it is the opposite), (d) that most of the OTUs found in the naturally exposed samples are highly related to species from marine/watery/saline environments, (e) that the majority of OTUs found in the artificially exposed tephra reflect the human activity around, (f) the higher naturally exposed TOC values, despite the higher water availability in the artificially exposed tephra, (g) the number of different biosignatures only detectable (or detectable in higher amounts) in the naturally exposed tephra, which among others, suggest an active metabolic state, and (h) that the Coastal Range of the Atacama is among the oldest formations of this desert (upper Triassic, 237 to 201.4 Mya)^86^, our collection of findings suggest that the inspected subsurface tephra contains some of the microorganisms that may be considered among the true native microorganisms of the Atacama (i.e. *Aquibacillus* sp. Atacama). Such species arrived from a saline environment (the Pacific Ocean and/or intertidal pools, in which salinity due to evaporation and UV radiation are higher during low tides) and then were selected, and then adapted in time, (having the advantage of producing compatible solutes)^76^ to tolerate an extremely dry environment^15^.

**Fig. 5:**
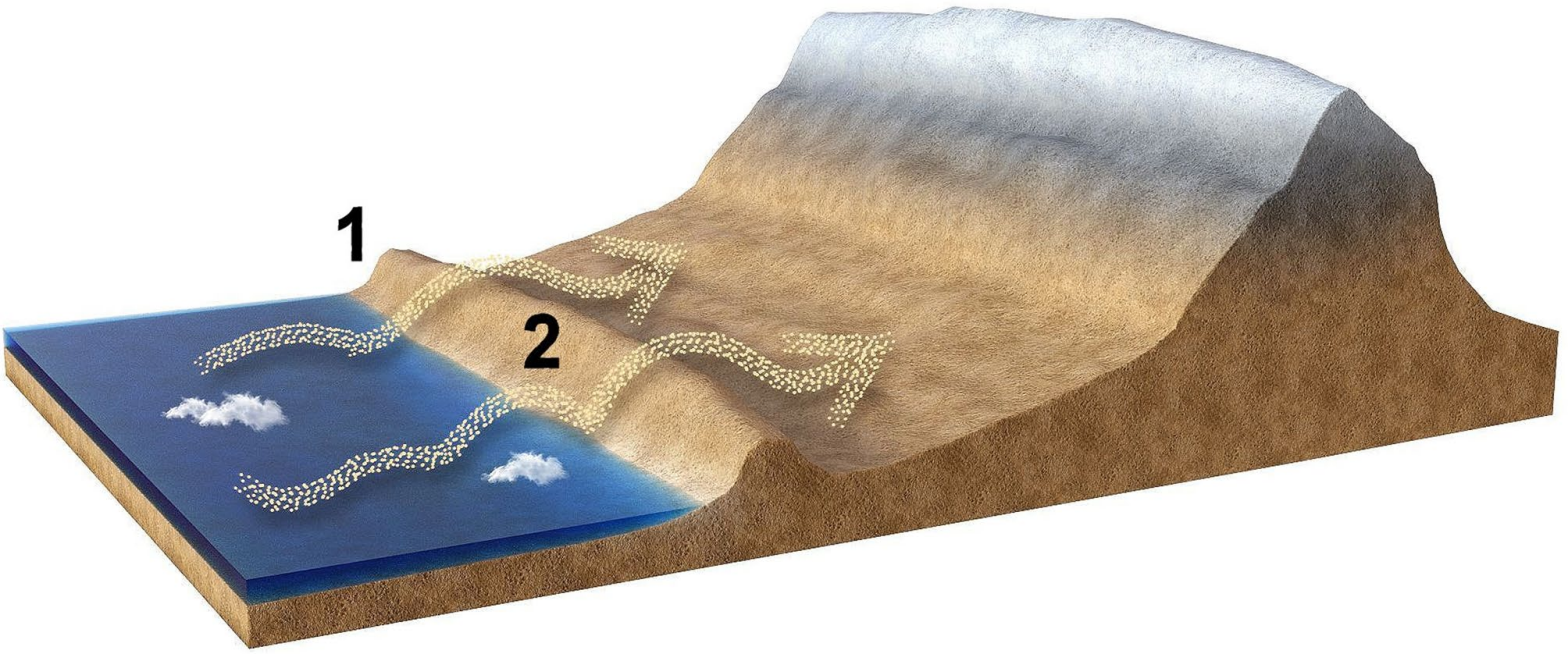
Model on how microbial life may have colonized Kenorland from the Panthalassic Ocean during the Neoarchaean. In the proposed model (as we have shown is taking place in the Coastal Range in front of the Atacama Desert), microbial life either directly goes inland from the ocean (1) or first arrive (2) and adapts to the coastal conditions (tidal pools or other places with lower availability of water), using wind transported dust. As shown in this report, most of these species will die in the process, but a few, having the molecular mechanisms that allowed them to survive high salinity, will be able to adapt, by exaptation, to the new conditions of low water availability imposed by land.

These statements back up our reported hypothesis that exaptation is the evolutionary mechanism that explain the microbial colonization of the driest desert on Earth^13^, in which the microbial species which already had some of the molecular mechanisms that allowed to adapt to low water activity caused by high salinity, along the transport mechanism (sea spray aerosols at the meters scale and wind transported dust at the kilometers scale), as evidenced here, provides an evolutionary and mechanistic model to understand how microbial life colonized the land from the sea in the Neoarchean (Fig. 5).

As Kenorland formed through the amalgamation of Archean cratons^87^, with plate tectonics (subduction and continental collision) still emerging^88^, the widespread basaltic volcanism of this era likely produced tephra similar to that inspected here. In addition, shallow marine shelves and volcanic islands were common, with sedimentary basins already hosting banded iron formations (BIFs), shales, and stromatolitic carbonates^89^.

Thus, considering that microbial life was widespread in shallow waters at that time (building stromatolites and driving the Neoarchaean atmospheric oxygen rise^90,91^, together with environmental changes that drastically distinguished the era by encouraging microbial metabolisms to evolve and diversify^92^, the Coastal Range of the Atacama may be considered an analog model for how life first colonized land from the ocean, with wind and exaptation as the main physical and evolutionary mechanisms.

## Materials and methods

### Sampling sites and dates

Mancha Blanca is located at 23º45’24.14’’S, 70º20’41.37’’W along route 28 that connect the coast of the Atacama with route 5. Samples and other analyses were taken in December of 2020, October of 2021, February of 2022 and August of 2022. Twelve tephra samples (six per exposure condition) were taken using sterile gloves and sterile 50 ml falcon tubes (Thermo Fisher Scientific, Massachusetts, USA). Care was taken to sample artificially exposed samples and naturally exposed samples separately, using separate collection tools. All samples were processed in the lab after one week after been collected under a UVC sterilized flood chamber, where equal amounts of each sample per exposure condition were collected and binned for cultivation and DNA extraction.

### Environmental characterization (temperature and relative humidity)

Temperature and relative humidity were measured in the field using dual Hygrochron temperature/Humidity micro data-loggers (Maxim Integrated, San Jose, CA, USA) as previously performed^8,9^, set to record data every 30 min during July 10 and August 6 of 2022. Sensors were inserted two centimeters below the surface of both naturally and artificially exposed tephra layers.

### X‑ray diffraction

Bulk tephra samples were stored at room temperature and then ground in the lab into powder.

with an agate mortar and pestle (Pulverisette 2, Fristsch, Idar-Oberstein, Germany), and X-ray powder diffraction data were collected using a Bruker D8 Eco Advance (Massachusetts, USA) in Bragg–Brentano geometry, Cu Kα radiation and Lynxeye XE-T linear detector. The X-ray generator was operated at 40 kV and 25 mA. Samples were scanned with a 0.05° (2θ) step size, over the range 5–60° (2θ) with a 1 s collection time at each step. Phase identification was performed by comparing the measured diffraction pattern (diffractograms) with patterns the PDF Database with the DIFFRAC.EVA software (Bruker AXS, Massachusetts, USA). https://www.bruker.com/es/products-and-solutions/diffractometers-and-x-ray-microscopes/x-ray-diffractometers/diffrac-suite-software/diffrac-eva.html.

### X-ray fluorescence

One gram of each sample was ground in an automatic agate mortar and compacted in a press. XRF analysis of major elements in the pressed samples were performed by the service provided by the SCT Unit of Oviedo University in Spain, using a Philips PW2404 X-ray fluorescence spectrometer, equipped with an PW2540 automatic charger.

### Raman spectroscopy

Raman spectra were performed exciting the sample with a non-polarized Nd: YAG solid state laser of 532 nm wavelength. After focusing onto a monochromator (Horiba Jobin Yvon HRi550), with a diffraction grating of 1200 grooves/mm, the scattered light was detected with a Charge Coupled Device (CCD), 1024 × 256 pixels, cooled to 203 K for thermal-noise reduction. The spectrometer is connected by fiber optics to a B&W Tek microscope with a 50x objective that allows a spot size on the sample of 42 μm (Microbeam S. A., Spain). The spectral resolution, with slit width of 200 μm, results better than 5 cm^−1^. Raman spectra were taken at laser power of 50 mW, and 10–100 s of integration times and 1–3 accumulations.

### DNA extraction from tephra samples

DNA extracted from the tephra samples were obtained in the lab was done by using the DNeasy PowerSoil Pro Kit (Quiagen, Düsseldorf, Germany) according to the manufacturer instructions, using a FastPrep-24 5G homogenizer (MP Biomedicals, Irvine, USA).

### Illumina NGS-Based sequencing and analyzes

Sequencing was performed at the facilities of Macrogen Inc (Seoul, South Korea). Libraries were prepared according to the Illumina 16 S Metagenomic Sequencing Library protocols to amplify the V3 and V4 region ITS3-ITS4 region and 18 S region The input gDNA 2ng was PCR amplified with 5x reaction buffer, 1mM of dNTP mix, 500nM each of the universal F/R PCR primer, and Herculase II fusion DNA polymerase (Agilent Technologies, Santa Clara, CA). Cycle conditions for the 1st´ PCR were; 3 min at 95 °C for heat activation, and 25 cycles of 30 s at 95 °C, 30 s at 55 °C and 30 s at 72 °C, followed by a 5-min final extension at 72 °C. The universal primer pair with Illumina adapter overhang sequences used for the first amplifications were as follows: 16 S Amplicon PCR Forward Primer.

5’ TCGTCGGCAGCGTCAGATGTGTATAAGAGACAGCCTACGGGNGGCWGCAG3’

And 16 S Amplicon PCR Reverse Primer

5’GTCTCGTGGGCTCGGAGATGTGTATAAGAGACAGGACTACHVGGGTATCTAATCC3’ (for bacteria). ITS3 Amplicon PCR Forward Primer

5’ TCGTCGGCAGCGTCAGATGTGTATAAGAGACAGGCATCGATGAAGAACGCAGC3’ and ITS4 Amplicon PCR Forward Primer

5’ GTCTCGTGGGCTCGGAGATGTGTATAAGAGACAGTCCTCCGCTTATTGATATGC3’ for fungi, and 18 S Amplicon PCR Forward Primer 5’TCGTCGGCAGCGTCAGATGTGTATAAGAGACAGGTACACACCGCCCGTC3´ and 18 S Amplicon PCR Forward Primer 5´ GTCTCGTGGGCTCGGAGATGTGTATAAGAGACAGTGATCCTTCTGCAGGTTCACCT3’ (for all eukaryotes). 1 st PCR products were purified with AMPure beads (Agencourt Bioscience, Beverly, MA). Following purification, 2ul of 1 st PCR products were PCR amplified for final library construction containing the index using NexteraXT Indexed Primer. The cycle conditions for the 2nd PCR were same as the 1 st PCR condition except that 10 amplification cycles less were used. PCR products were purified with AMPure beads. The final purified products were then quantified using qPCR according to the qPCR Quantification Protocol Guide (KAPA Library Quantificatoin kits for Illumina Sequecing platforms) and their quality checked using the TapeStation D1000 ScreenTape (Agilent Technologies, Waldbronn, Germany), and then sequenced using the MiSeq™ platform (Illumina, San Diego, USA).

The resulting raw sequences were processed in MOTHUR software (v.1.43.0, https://mothur.org/) using a custom script based upon MiSeq SOP that maximizes sequence accuracy by restrictive quality thresholds at several steps. The resulting identity OTUs sequences were then manually checked using the Megablast option for highly similar sequences (only > 95% sequence similarity was accepted) of the BLASTN algorithm against the National Centre for Biotechnology Information nonredundant database (www.ncbi.nlm.nih.gov*).*

### Cultivation of isolates from tephra samples

Tephra samples were stored in the lab at room temperature, and then aseptically inoculated in Petri dishes containing agar and either Luria–Bertani Broth (Sigma-Aldrich, Missouri, USA) Marine Media (CondaLab, Torrejón de Ardoz, Spain) or R2A media (Condalab, Torrejón de Ardoz, Spain). Colonies arising from tephra particles usually were evident two weeks after inoculation. These colonies were then re-cultivated in the media from they were first isolated, to obtain enough biomass for DNA extraction and storage.

### DNA extraction from isolates

DNA extracted from the colonies obtained in the lab was done by using the DNeasy UltraClean Microbial Kit (Quiagen, Düsseldorf, Germany) according to the manufacturer instructions, using a FastPrep-24 5G homogenizer (MP Biomedicals, Irvine, USA).

### 16S rRNA amplification and sequencing from isolates DNA

16 S rRNA of bacterial isolates was amplified in the lab using the GoTaq Green Master Mix (Promega, Wisconsin, USA) and the primers 341f (5′CCT ACG GGNGGC WGC AG3′) and 785r (5′GAC TAC HVGGG TAT CTA ATC C′). PCR conditions used were: 95 °C for 5 min, and 25 cycles of (95 °C for 40 s, 55 C for 2 min, 72 °C for 1 min) followed by 72 °C for 7 min. The resultant reactions were visualized in a 1.5% agarose TAE gel at 50 V. The automated sequencing of the resulting PCR products was conducted by Macrogen DNA Sequencing Inc. (Seoul, Korea). Sequences were checked for quality using the BioEdit software (Ibis Therapeutics, Carlsbad, USA) and end-trimmed before using the Megablast option for highly similar sequences of the BLASTN algorithm against the National Centre for Biotechnology Information nonredundant database (www.ncbi.nlm.nih.gov) to search for the closest species of each of the isolates obtained. Only species with at least 98% of sequence identify and an E value of 0.0 were selected, and only species with defined genus and species names were considered for phylogenetic closeness.

### Phylogenetic analysis of isolates

In the lab, phylogenetic analysis of 16 S rRNA gene sequences obtained from isolates were aligned by multiple sequence comparison by log-expectation^93^, analyzed with jModelTest^94^ and then by Phylip NJ^95^ (bootstrap 1000), all tools of the freely available Bosque phylogenetic analysis software (version 1.7.152)^95^. All sequences are available in GenBank; https://submit.ncbi.nlm.nih.gov/subs/genbank/SUB15688376.

### Biosignature analyses

#### Isotopic analysis

Stable isotopic composition of organic carbon (δ^13^C) and total nitrogen (δ^15^N) was measured on the bulk sediment samples using isotope-ratio mass spectrometry (IRMS), following the United States Geological Survey method. In the first place, samples were homogenized by manual grinding in a mortar. Then, they were decarbonated with HCl (3 N) and, after 24 h of equilibration, they were adjusted to neutral pH with ultrapure water. Later, the samples were dried in an oven at 50 °C until constant weight. Ratios of δ^13^C and δ^15^N were measured in a MAT 253 IRMS (Thermo Fisher Scientific, Waltham, Massachusetts, USA) and reported in the standard per mil notation (‰). Three certified standards were used (USGS41, IAEA-600, and USGS40) with an analytical precision of 0.1‰. The content of total nitrogen (TN %) and total organic carbon (TOC %) was measured with an elemental analyzer (HT Flash, Thermo Fisher Scientific, Waltham Massachusetts, USA) during measurement of the stable isotopes.

#### Extraction and analysis of lipid biomarkers

Lipids of six lyophilized and ground subsamples (8–15 g) of Mancha Blanca samples were extracted with ultrasound sonication (3 × 15 min) using 15 ml of a 3:1 (v/v) mixture of dichloromethane (DCM) and methanol (MeOH) to obtain a 45 ml of TLE. Before the extraction, tetracosane-D50, myristic acid-D27 and 2-hexadecanol were added as internal standards.

The concentrated and desulfurized TLE were hydrolyzed overnight with KOH (6% MeOH) at room temperature. Then, a liquid-liquid extraction with *n*-hexane (3 × 30 ml) was performed to recover the neutral fraction and acidification with HCl (37%) were employed to separate the acidic fraction of the hydrolyzed TLE. Further separation of the neutral fraction into non-polar (hydrocarbons) and polar (alkanols and sterols) was done according to a method described in detail elsewhere^96^. The acidic fraction was transesterified with BF3 in MeOH to produce fatty acid methyl esters (FAMEs), and the polar fraction was trimethylsilylated (N, O-bis [tri- methylsilyl] trifluoroacetamide [BSTFA]) to analyze the resulting trimethyl silyl alkanols.

All fractions were analyzed using gas chromatography-mass spectrometry (GC–MS) using a 8860 GC System coupled to a 5977B MSD (Agilent Technologies, Santa Clara, CA, USA) operating with electron ionization at 70 eV and scanning from m/z 50 to 650. Compound identification was based on retention time and mass spectra comparison with reference materials and the NIST mass spectral database. Quantification was performed with the use of external calibration curves of n-alkanes (C_10_ to C_40_), FAMEs (C_6:0_ to C_24:0_) and *n*-alkanols (C_14_, C_18_ and C_20_), all supplied by Sigma-Aldrich (Madrid, Spain). Recovery of the internal standards were measured to average 74 ± 13%.

### Bright field and fluorescence microscopy

Cells of the *Aquibacillus* sp Atacama isolate were grown in marine media and then resuspended in milli-q water. Observations were made in differential interference contrast (DIC) using a Zeiss AXIO Imager M2 fluorescence microscope (Carl Zeiss, Jena, Germany) and an Apochrome × 60, *n* = 1.4 Zeiss oil-immersion objective. A CCD Axiocam HRc Rev 2camera and AXIOVISION software (Version 4.7, Carl Zeiss, Oberkochen, Germany, https://www.micro-shop.zeiss.com/en/us/system/software-axiovision+software-products/1007/). were used to capture and record images.

## Supplementary Information

Below is the link to the electronic supplementary material.

Supplementary Material 1

## Data Availability

The authors declare that all the data supporting the findings of this study are available within the article (and its Supplementary Information file), or available from the corresponding authors on reasonable request.

## References

[1] Horodyski, R. J. & Knauth, L. P. Life on land in the precambrian. *Science* **263**, 494–498 (1994). 17754880 10.1126/science.263.5146.494

[2] Watanabe, Y. et al. Geochemical evidence for terrestrial ecosystems 2.6 billion years ago. *Nature* **408**, 574–578 (2000). 11117742 10.1038/35046052

[3] Mojzsis, S. J. et al. Evidence for life on Earth before 3,800 million years ago. *Nature* **384**, 55–59 (1996). 8900275 10.1038/384055a0

[4] Azua-Bustos, A. et al. Life at the dry edge: microorganisms of the Atacama desert. *FEBS Lett.* **586**, 2939–2945 (2012). 22819826 10.1016/j.febslet.2012.07.025

[5] Azua-Bustos, A. et al. Ancient photosynthetic eukaryote biofilms in an Atacama desert coastal cave. *Microb. Ecol.* **58**, 485–496 (2009). 19259626 10.1007/s00248-009-9500-5

[6] Azua-Bustos, A. et al. A novel subaerial Dunaliella sp. Growing on cave spiderwebs in the Atacama desert. *Extremophiles* **14**, 443–452 (2010). 20623153 10.1007/s00792-010-0322-7

[7] Azua-Bustos, A. et al. Hypolithic cyanobacteria supported mainly by fog in the coastal range of the Atacama desert. *Microb. Ecol.* **61**, 568–581 (2011). 21188376 10.1007/s00248-010-9784-5

[8] Azua-Bustos, A. et al. Gloeocapsopsis AAB1, an extremely desiccation tolerant Cyanobacterium isolated from the Atacama desert. *Extremophiles* **18**, 61–74 (2014). 24141552 10.1007/s00792-013-0592-y

[9] Puente-Sánchez, F. et al. Draft genome sequence of the extremely Desiccation-Tolerant Cyanobacterium Gloeocapsopsis sp. Strain AAB1. *Genome Announc*. **6**, 216–218 (2018). 10.1128/genomeA.00216-18 PMC5920166 29700137

[10] Jung, P. et al. Diffluens Sp. Nov. And G. Dulcis Sp. Nov. Isolated from the coastal range of the Atacama desert (Chile). *Front. Microbiol.* **12**, 671742 (2021). 34305839 10.3389/fmicb.2021.671742 PMC8295473

[11] Moore, R. A. et al. Unveiling metabolic pathways involved in the extreme desiccation tolerance of an Atacama Cyanobacterium. *Sci. Rep.* **13**, 15767 (2023). 37737281 10.1038/s41598-023-41879-8 PMC10516996

[12] Azua-Bustos. Gloeocapsopsis AAB1, A Cyanobacterium Highly Tolerant To Desiccation Isolated From The Atacama Desert. Doctoral Thesis, Pontificia Universidad Católica de Chile (2013).

[13] Azua-Bustos, A. et al. Extreme environments as potential drivers of convergent evolution by exaptation: the Atacama desert coastal range case. *Front. Microbiol.* **3**, 426 (2012). 23267354 10.3389/fmicb.2012.00426 PMC3526103

[14] Gould, S. J. & Vrba, E. S. Exaptation—a missing term in the science of form. *Paleobiology* **8**, 4–15 (1982).

[15] Azua-Bustos, A. et al. Aeolian transport of viable microbial life on a Mars analog environment. *Implications Mars Sci. Rep.* **9**, 1–11 (2019). 10.1038/s41598-019-47394-z PMC6706390 31439858

[16] Azua-Bustos, A. et al. Sea spray allows for the growth of subaerial microbialites at the driest desert on Earth. *Sci. Rep.* **14**, 19915 (2024). 39198637 10.1038/s41598-024-70447-x PMC11358262

[17] Ministerio de Obras Públicas del SEREMI de la Región de Antofagasta. Retrieved in August 11, (2022). http://antofagasta.mop.cl/noticias/Paginas/DetalledeNoticias.aspx?item=68

[18] González, G., & Niemeyer, H. Cartas Antofagasta y Punta Tetas, Región de Antofagasta. Servicio Nacional de Geología y Minería, Carta Geológica de Chile, Serie Geología Básica, No. 89, 35 p., 1 mapa escala 1:100.000 (2005).

[19] Naranjo, J. A. Interpretación de La actividad cenozoica superior a Lo Largo de La Zona de falla de Atacama, Norte de Chile. *Rev. Geol. Chile*. **31**, 43–55 (1987).

[20] Marinovic, N., et. al. Hoja Aguas Blancas, Región de Antofagasta. Servicio Nacional de Geología y Minería, Carta Geológica de Chile, No. 70, escala 1:250.000 (1995).

[21] Carrizo, D.A. La deformación neógeno – cuaternaria del sistema de fallas de atacama, en el borde oriental de la cordillera de la costa de Antofagasta, norte de Chile. Doctoral Thesis manuscript presented at the Universidad Católica del Norte, Chile (2002).

[22] Azua-Bustos, A. Discovery and microbial content of the driest site of the hyperarid Atacama desert. *Chile Environ. Microbiol. Rep.* **7**, 388–394 (2015). 25545388 10.1111/1758-2229.12261

[23] Chen, S. Haloparvum sedimenti gen. nov., sp. nov., a member of the family haloferacaceae. *Int. J. Syst. Evo L Microbiol.* **66**, 2327–2334 (2016). 10.1099/ijsem.0.001033 27001607

[24] Jingjing, L. Swionibacillus sediminis gen. nov., sp. nov., a member of the family bacillaceae isolated from ocean sediment. *Int. J. Syst. Evol. Microbiol.* **67**, 3440–3445 (2017). 28857029 10.1099/ijsem.0.002133

[25] Amoozegar, M. A. Aquibacillus halophilus gen. Nov., sp. Nov., a moderately halophilic bacterium from a hypersaline lake, And reclassification of Virgibacillus Koreensis as Aquibacillus Koreensis comb. Nov. And Virgibacillus albus as Aquibacillus albus comb. Nov. *Int. J. Syst. Evol. Microbiol.* **64**, 3616–3623 (2014). 25062698 10.1099/ijs.0.065375-0

[26] Albuquerque, L. Bacillus Isabeliae sp. nov., a halophilic bacterium isolated from a sea salt evaporation pond. *Int. J. Syst. Evol. Microbiol.* **58**, 226–230 (2008). 18175713 10.1099/ijs.0.65217-0

[27] Senghor, B. Description of ‘Bacillus dakarensis’ sp. nov., ‘Bacillus sinesaloumensis’ sp. nov., ‘Gracilibacillus timonensis’ sp. nov., ‘Halobacillus massiliensis’ sp. nov., ‘Lentibacillus massiliensis’ sp. nov., ‘Oceanobacillus senegalensis’ sp. nov., ‘Oceanobacillus timonensis’ sp. nov., ‘Virgibacillus dakarensis’ sp. nov. And ‘Virgibacillus marseillensis’ sp. nov., nine halophilic new species isolated from human stool. *New. Microbes New. Infect.* **17**, 45–51 (2017). 28280541 10.1016/j.nmni.2017.01.010 PMC5333509

[28] Zhang, M. Y. Bacillus Notoginsengisoli sp. nov., a novel bacterium isolated from the rhizosphere of Panax Notoginseng. *Int. J. Syst. Evol. Microbiol.* **67**, 2581–2585 (2017). 28786779 10.1099/ijsem.0.001975

[29] Ruiz-García, C. Bacillus velezensis sp. nov., a surfactant-producing bacterium isolated from the river Vélez in Málaga, Southern Spain. *Int. J. Syst. Evol. Microbiol.* **55**, 191–195 (2005). 15653875 10.1099/ijs.0.63310-0

[30] Watanabe, M., et. al. Limnochorda pilosa gen. nov., sp. nov., a moderately thermophilic, facultatively anaerobic, pleomorphic bacterium and proposal of Limnochordaceae fam. nov., Limnochordales ord. nov. and Limnochordia classis nov. in the phylum Firmicutes. *Int. J. Syst. Evol. Microbiol.* 2015. **65**:2378–2384 (2015). 10.1099/ijs.0.000267 25896353

[31] Yumoto, I. Bacillus horti sp. nov., a new gram-negative alkaliphilic Bacillus. *Int. J. Syst. Bacteriol.* **8**, 565–571 (1998). 10.1099/00207713-48-2-565 9731298

[32] Nuñes, I., Al & et. Paucisalibacillus globulus gen. nov., sp. nov., a Gram-positive bacterium isolated from potting soil. *Int. J. Syst. Evol. Microbiol.* **56**, 1841–1845 (2006). 16902018 10.1099/ijs.0.64261-0

[33] Shih-Yao, L. Ammoniphilus resinae sp. nov., an endospore-forming bacterium isolated from resin fragments. *Int. J. Syst. Evol. Microbiol.* **66**, 3010–3016 (2016). 27151144 10.1099/ijsem.0.001137

[34] Patel Sudip, P. & Rhadey, G. A phylogenomic And comparative genomic framework for resolving the polyphyly of the genus bacillus: proposal for six new genera of Bacillus species, Peribacillus gen. Nov., Cytobacillus gen. Nov., Mesobacillus gen. Nov., Neobacillus gen. Nov., Metabacillus gen. Nov. And Alkalihalobacillus gen. Nov. *Int. J. Syst. Evol. Microbiol.* **70**, 406–438 (2020). 31617837 10.1099/ijsem.0.003775

[35] Nogi, Y. Characterization of alkaliphilic Bacillus strains used in industry: proposal of five novel species. *Int. J. Syst. Evol. Microbiol.* **55**, 2309–2315 (2005). 16280488 10.1099/ijs.0.63649-0

[36] Ouyang, T. H. et. al. Caldalkalibacillus Salinus sp. nov., isolated from a salt lake in Xinjiang, Northwest China. *Arch. Microbiol.* **204**, 179 (2022). 35174423 10.1007/s00203-022-02789-x

[37] X & Wu Paenibacillus tianmuensis sp. nov., isolated from soil. *Int. J. Syst. Evol. Microbiol.* **61**, 1133–1137 (2011). 20543152 10.1099/ijs.0.024109-0

[38] Kuroshima, K. Bacillus Ehimensis sp. nov. And Bacillus chitinolyticus sp. nov., new chitinolytic members of the genus Bacillus. *J. Syst. Evol. Microbiol.* **46**, 76–80 (1996).

[39] Yahiaoui, M. Purification and biochemical characterization of a new organic solvent-tolerant chitinase from Paenibacillus timonensis strain LK-DZ15 isolated from the Djurdjura mountains in Kabylia, Algeria. *Carbohydr. Res.* **483**, 107747 (2019). 31349143 10.1016/j.carres.2019.107747

[40] Ormerod, J. G. Heliophilum fasciatum gen. nov. sp. nov. And Heliobacterium gestii sp. nov.: endospore-forming heliobacteria from rice field soils. *Arch. Microbiol.* **165**, 226–234 (1996). 8952943 10.1007/s002030050320

[41] Shin, S. K. et. al. Paenibacillus crassostreae sp. nov., isolated from the Pacific oyster Crassostrea gigas. *Int. J. Syst. Evol. Microbiol.* **68**, 58–63 (2018). 29068277 10.1099/ijsem.0.002444

[42] Tamura, T. Description of actinomycetospora chibensis sp. nov., actinomycetospora Chlora sp. nov., actinomycetospora cinnamomea sp. nov., actinomycetospora corticicola sp. nov., actinomycetospora lutea sp. nov., actinomycetospora straminea sp. nov. And actinomycetospora succinea sp. nov. And emended description of the genus actinomycetospora. *Int. J. Syst. Evol. Microbiol.* **61**, 1275–1280 (2011). 20622052 10.1099/ijs.0.024166-0

[43] Van Pham, H. T. & Kim, J. Bacillus thaonhiensis sp. nov., a new species, was isolated from the forest soil of Kyonggi university by using a modified culture method. *Curr. Microbiol.* **68**, 88–95 (2014). 23995763 10.1007/s00284-013-0443-1

[44] Mongaret, C. Cutibacterium acnes: the urgent need to identify diagnosis markers. *Infect. Immun.* **89**, e00753–e00720 (2021). 33468579 10.1128/IAI.00753-20 PMC8090951

[45] Natsis, N. & Cohen, P. Coagulase-Negative Staphylococcus skin and soft tissue infections. *Am. J. Clin. Dermatol.* **19**, 671–677 (2018). 29882122 10.1007/s40257-018-0362-9

[46] Wolfrum, A. Granulomatous mastitis: A therapeutic and diagnostic challenge. *Breast Care (Basel)*. **13**, 413–418 (2018). 30800035 10.1159/000495146 PMC6381909

[47] Veloo, A. C. M. Anaerococcus Nagyae sp. nov., isolated from human clinical specimens. *Anaerobe* **38**, 111–115 (2016). 26639871 10.1016/j.anaerobe.2015.11.009

[48] Padmanabhan, R. et al. Genome sequence and description of Corynebacterium ihumii sp. nov. *Stand. Genomic Sci.* **9**, 1128–1143 (2014). 25197488 10.4056/sigs.5149006 PMC4149009

[49] Lee, S. D. Jiangella alkaliphila sp. nov., an actinobacterium isolated from a cave. *Int. J. Syst. Evol. Microbiol.* **58**, 1176–1179 (2008). 18450709 10.1099/ijs.0.65479-0

[50] Vaneechoutte, M. et al. Chryseobacterium hominis sp. nov., to accommodate clinical isolates biochemically similar to CDC groups II-h and II-c. *Int. J. Syst. Evol. Microbiol.* **57**, 2623–2628 (2007). 17978230 10.1099/ijs.0.65158-0

[51] Cools, P. Atopobium deltae sp. nov., isolated from the blood of a patient with fournier’s gangrene. *Int. J. Syst. Evol. Microbiol.* **64**, 3140–3145 (2014). 24944340 10.1099/ijs.0.065243-0

[52] McIlvanna, E. Fusobacterium nucleatum and oral cancer: a critical review. *BMC Cancer*. **21**, 1212 (2021). 34774023 10.1186/s12885-021-08903-4 PMC8590362

[53] Duncan, S. H. et. al. Proposal of roseburia faecis sp. nov., roseburia hominis sp. nov. And roseburia inulinivorans sp. nov., based on isolates from human faeces. *Int. J. Syst. Evol. Microbiol.* **56**, 2437–2441 (2006). 17012576 10.1099/ijs.0.64098-0

[54] Merten, M. Complete genome sequence and annotation of Corynebacterium singulare DSM 44357, isolated from a human semen specimen. *Genome Announc*. **3**, e00183–e00115 (2015). 25814602 10.1128/genomeA.00183-15 PMC4384142

[55] Tabbuso, T. Moraxella Osloensis infection among adults and children: A pediatric case and literature review. *Arch. Pediatr.* **28**, 348–351 (2021). 33858729 10.1016/j.arcped.2021.03.003

[56] Dekio, I. Cutibacterium modestum sp. nov., isolated from Meibum of human meibomian glands, and emended descriptions of Cutibacterium granulosum and Cutibacterium Namnetense. *Int. J. Syst. Evol. Microbiol.* **70**, 2457–2462 (2020). 32559834 10.1099/ijsem.0.004058

[57] Ramesh, R. Lawsonella clevelandensis: an emerging cause of vascular graft infection. *BMJ Case Rep.* **14**, e237350 (2021). 33637490 10.1136/bcr-2020-237350 PMC7919573

[58] Takahashi, N. & Yamada, T. Glucose and lactate metabolism by actinomyces Naeslundii. *Crit. Rev. Oral Biol. Med.* **10**, 487–503 (1999). 10634585 10.1177/10454411990100040501

[59] Korcari, D. et al. Exploration of Lactiplantibacillus fabifermentans and Furfurilactobacillus Rossiae as potential cocoa fermentation starters. *J. Appl. Microbiol.* **133**, 1769–1780 (2022). 35751485 10.1111/jam.15687 PMC9540988

[60] Wang, Y. S. et. al. Paracoccus pueri sp. nov., isolated from pu’er tea. *Antonie Van Leeuwenhoek*. **111**, 1535–1542 (2018). 29484518 10.1007/s10482-018-1041-9

[61] Falagán, C. & Johnson, D. B. Acidibacter ferrireducens gen. nov., sp. nov.: an acidophilic ferric iron-reducing gammaproteobacterium. *Extremophiles* **18**, 1067–1073 (2014). 25116055 10.1007/s00792-014-0684-3

[62] Oh, Y. J. et. al. Virgibacillus Kimchii sp. nov., a halophilic bacterium isolated from Kimchi. *J. Microbiol.* **55**, 933–938 (2017). 29214493 10.1007/s12275-017-7386-3

[63] Jia, J. Phenotype profiles and adaptive preference of acinetobacter Johnsonii isolated from Ba river with different environmental backgrounds. *Environ. Res.* **196**, 110913 (2021). 33639142 10.1016/j.envres.2021.110913

[64] Cobo, F. Bacteremia caused by Veillonella dispar in an oncological patient. *Anaerobe* **66**, 102285 (2020). 33075505 10.1016/j.anaerobe.2020.102285 PMC7563575

[65] Park, S. Rubrivirga Marina gen. nov., sp. nov., a member of the family Rhodothermaceae isolated from deep seawater. *Int. J. Syst. Evol. Microbiol.* **63**, 2229–2233 (2013). 23148103 10.1099/ijs.0.046318-0

[66] Bouju-Albert, A. Quantification of viable brochothrix thermosphacta in Cold-Smoked salmon using PMA/PMAxx-qPCR. *Front. Microbiol.* **12**, 654178 (2021). 34335490 10.3389/fmicb.2021.654178 PMC8316974

[67] Candeliere, F. Comparative genomics of leuconostoc carnosum. *Front. Microbiol.* **11**, 605127 (2021). 33505375 10.3389/fmicb.2020.605127 PMC7829361

[68] Hilgarth, M. Photobacterium carnosum sp. nov., isolated from spoiled modified atmosphere packaged poultry meat. *Syst. Appl. Microbiol.* **41**, 44–50 (2018). 29279139 10.1016/j.syapm.2017.11.002

[69] Liao, L. Complete genome sequence of Marinomonas Arctica BSI20414, a giant antifreeze protein-producing bacterium isolated from Arctic sea ice. *Mar. Genomics*. **57**, 100829 (2021). 33867119 10.1016/j.margen.2020.100829

[70] Sakdapetsiri, C. Paeniglutamicibacter terrestris sp. nov., isolated from phenanthrene-degrading consortium enriched from Antarctic soil. *Int. J. Syst. Evol. Microbiol.* **71**, 3 (2019). 10.1099/ijsem.0.004689 33555249

[71] Yu, Q. Rheinheimera sediminis sp. nov., a marine bacterium isolated from coastal sediment. *Int. J. Syst. Evol. Microbiol.* **70**, 1282–1287 (2020). 31800389 10.1099/ijsem.0.003917

[72] Park, E. J. et. al. Kocuria atrinae sp. nov., isolated from traditional Korean fermented seafood. *Int. J. Syst. Evol. Microbiol.* **60**, 914–918 (2010). 19661502 10.1099/ijs.0.014506-0

[73] Lee, S. W. Methylobacterium Dankookense sp. nov., isolated from drinking water. *J. Microbiol.* **47**, 716–720 (2009). 20127465 10.1007/s12275-009-0126-6

[74] Wang, H. Comparative proteomic insights into the lactate responses of halophilic salinicoccus roseus W12. *Sci. Rep.* **5**, 13776 (2015). 26358621 10.1038/srep13776 PMC4566078

[75] Ding, W. J. Aquibacillus rhizosphaerae sp. nov., an Indole acetic acid (IAA)-producing halotolerant bacterium isolated from the rhizosphere soil of Kalidium cuspidatum. *Curr. Microbiol.* **80**, 404 (2023). 37930394 10.1007/s00284-023-03543-2

[76] Empadinhas, N. & da Costa, M. S. Osmoadaptation mechanisms in prokaryotes: distribution of compatible solutes. *Int. Microbiol.* **11**, 151–161 (2008). 18843593

[77] Didyk, B. M. et. al. Organic geochemical indicators of palaeoenvironmental conditions of sedimentation. *Nature* **272**, 216–222 (1978).

[78] Brocks, J. J. & Summons, R. E. Sedimentary Hydrocarbons, biomarkers for early life. *Treatise Geochem.* **8–9**, 63–115 (2003).

[79] Peters K.E., et. al. The Biomarker Guide—Part II—Biomarkers and Isotopes in Petroleum Exploration and Earth History. Cambridge University Press, New York (2005).

[80] Tornabene, T. G. et. al. Squalenes, phytanes and other isoprenoids as major neutral lipids of methanogenic and thermoacidophilic archaebacteria. *J. Mol. Evol.* **13**, 3–83 (1979). 10.1007/BF01732755 458874

[81] Stiehl, T. et al. Molecular and isotopic characterization of lipids in cultured halophilic microorganisms from the dead sea and comparison with the sediment record of this hypersaline lake. *Org. Geochem.* **36**, 1242–1251 (2005).

[82] Ourisson, G. M. et al. Prokaryotic hopanoids and other polyterpenoid sterol surrogates. *Annu. Rev. Microbiol.* **41**, 301–333 (1987). 3120639 10.1146/annurev.mi.41.100187.001505

[83] Tobias, H. & Soffer, A. Chemisorption of halogen on carbons—I Stepwise chlorination and exchange of C-CI with CH bonds. *Carbon* **23**, 281–289 (1985).

[84] Fernandes, M. F. et al. Comparison of whole-cell fatty acid (MIDI) or phospholipid fatty acid (PLFA) extractants as biomarkers to profile soil microbial communities. *Microb. Ecol.* **66**, 145–157 (2013). 23443903 10.1007/s00248-013-0195-2

[85] Hartley, A. J. et al. ‎150 million years of Climatic stability: evidence from the Atacama Desert, Northern Chile. *J. Geol. Soc.* **62**, 421–424 (2005).

[86] Jara, J. J. Geochronology and petrogenesis of intrusive rocks in the coastal cordillera of Northern chile: insights from Zircon U-Pb dating and trace element geochemistry. *Gondwana Res.* **93**, 48–72 (2021).

[87] Yakubchuk, A. S. From Kenorland to modern continents: tectonics and metallogeny. *Geotectonics* **53**, 169–192 (2019).

[88] Hawkesworth, C. J., Cawood, P. A. & Dhuime, B. The evolution of the continental crust and the onset of plate tectonics. Front. Earth Sci. **8**, 262 (2020). 10.3389/feart.2020.00326 PMC7116083 32944569

[89] Xiang, W. et al. Were early Archean carbonate factories major carbon sinks on the juvenile Earth? *Biogeosciences* **24**, 5653–5684 (2024).

[90] Des Marais, D. J. Microbial mats, stromatolites and the rise of oxygen in the precambrian atmosphere. *Glob Planet. Change*. **97**, 93–96 (1991). 11538094

[91] Lepot, K. Signatures of early microbial life from the archean (4 to 2.5 Ga) eon. *Earth Sci. Rev.* **209**, 103296 (2020).

[92] Stüeken, E. E. et al. Environmental niches and metabolic diversity in neoarchean lakes. *Geobiology* **15**, 767–783 (2017). 28856796 10.1111/gbi.12251

[93] Edgar, R. C. MUSCLE: multiple sequence alignment with high accuracy and high throughput. *Nucleic Acids Res.* **32**, 1792–1797 (2004). 10.1093/nar/gkh340 PMC390337 15034147

[94] Posada, D. jModelTest: phylogenetic model averaging. *Mol. Biol. Evol.* **7**, 1253–1256 (2008). 10.1093/molbev/msn083 18397919

[95] Ramírez-Flandes, S. & Ulloa, O. Bosque: integrated phylogenetic analysis software. *Bioinformatics* **24**, 2539–2541 (2008). 10.1093/bioinformatics/btn466 18762483

[96] Sánchez-García, L. et al. Fingerprinting molecular and isotopic biosignatures on different hydrothermal scenarios of Iceland, an acidic and sulfur-rich Mars analog. *Sci. Rep.* **10**, 21196 (2020). 10.1038/s41598-020-78240-2 PMC7712778 33273669

