# Supplementary material for: An Atacama subsurface tephra layer reveals how life colonized Kenorland in the Neoarchean
Source: Sci Rep. 2025 Dec 9;15:43474. doi: 10.1038/s41598-025-24288-x (PMC12690102; doi:10.1038/s41598-025-24288-x)
Supplement: Supplementary file 1 — Supplementary Material 1 [file 41598_2025_24288_MOESM1_ESM.pdf]

Supplementary Files

An Atacama subsurface tephra unveil how life colonized Kenorland in the Neoarchean

Armando Azua-Bustos, Carlos González-Silva, Daniel Carrizo, Laura Sánchez-García, Maite Fernández-Sampedro, Thanh Quy Dang, Cristian Vargas-Carrera, Victoria Muñoz-Iglesias, María Paz Martin-Redondo, Pedro Mustieles-del-Ser, Olga Prieto-Ballesteros, Jacek Wierzchos.

Supplementary Figures 1 to 8

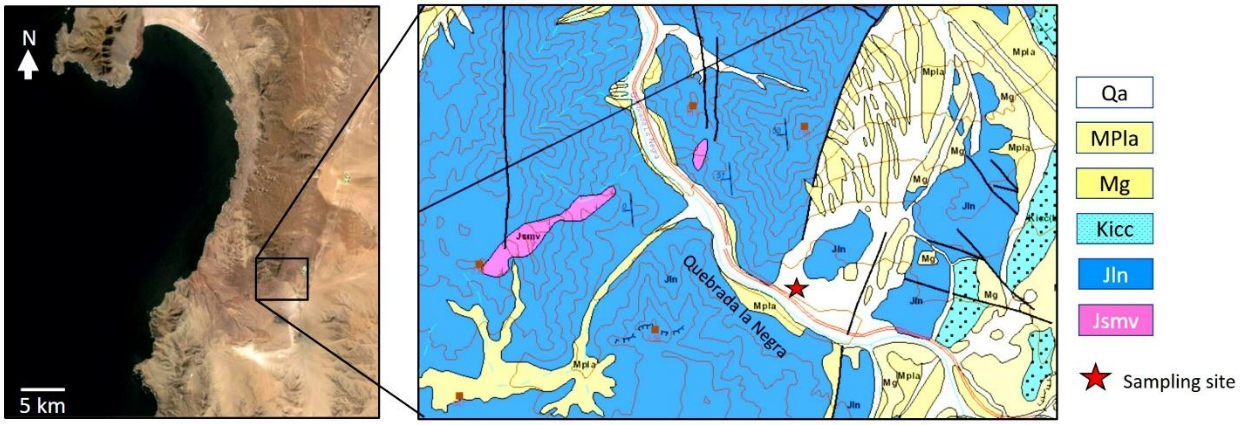

Figure S1. Geological context of the Mancha Blanca site.
Qa: Modern alluvial deposits. Mpla: Quaternary clays, sands, gravels (Ancient alluvial deposits). Mg: Miocene sands and gravels (Ancient alluvial deposits). Kicc: Miocene gravels of the Caleta Coloso Formation. JIn: Cretaceous conglomerates of La Negra Formation. Jsmv: Jurassic Formation. The satellite image shown in Panel A was obtained from Google Earth Pro, Version 7.3.6. The geological map on panel B was acquired at the online store of SERNAGEOMIN, Mapa Geológico de Chile 2002 (Carta Geológica de Chile, Serie Geología Básica nº075).

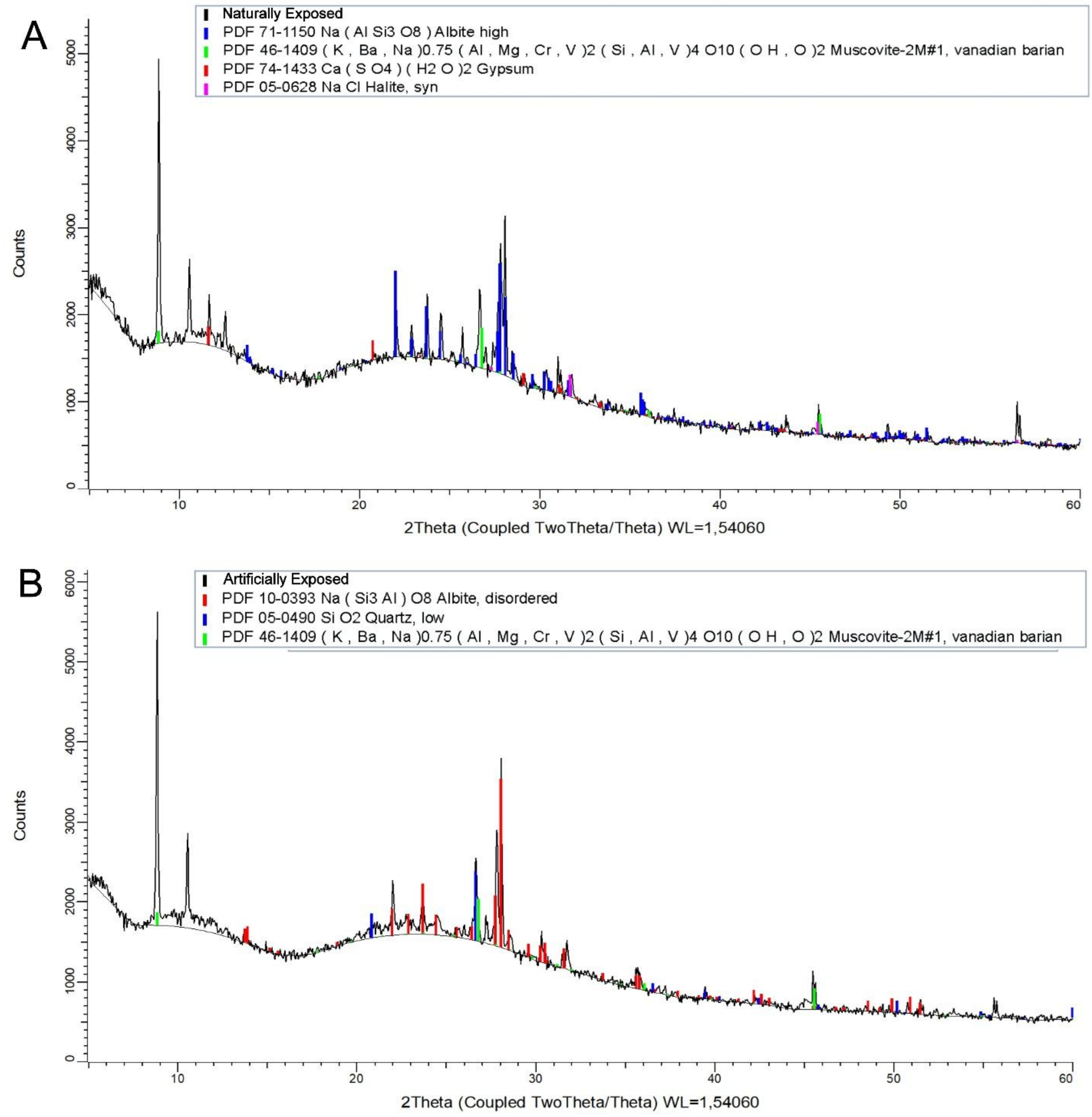

Figure S2.- X-Ray diffraction (XRD) patterns of Mancha Blanca samples. Minerals were determined by XRD, as detailed in methods.

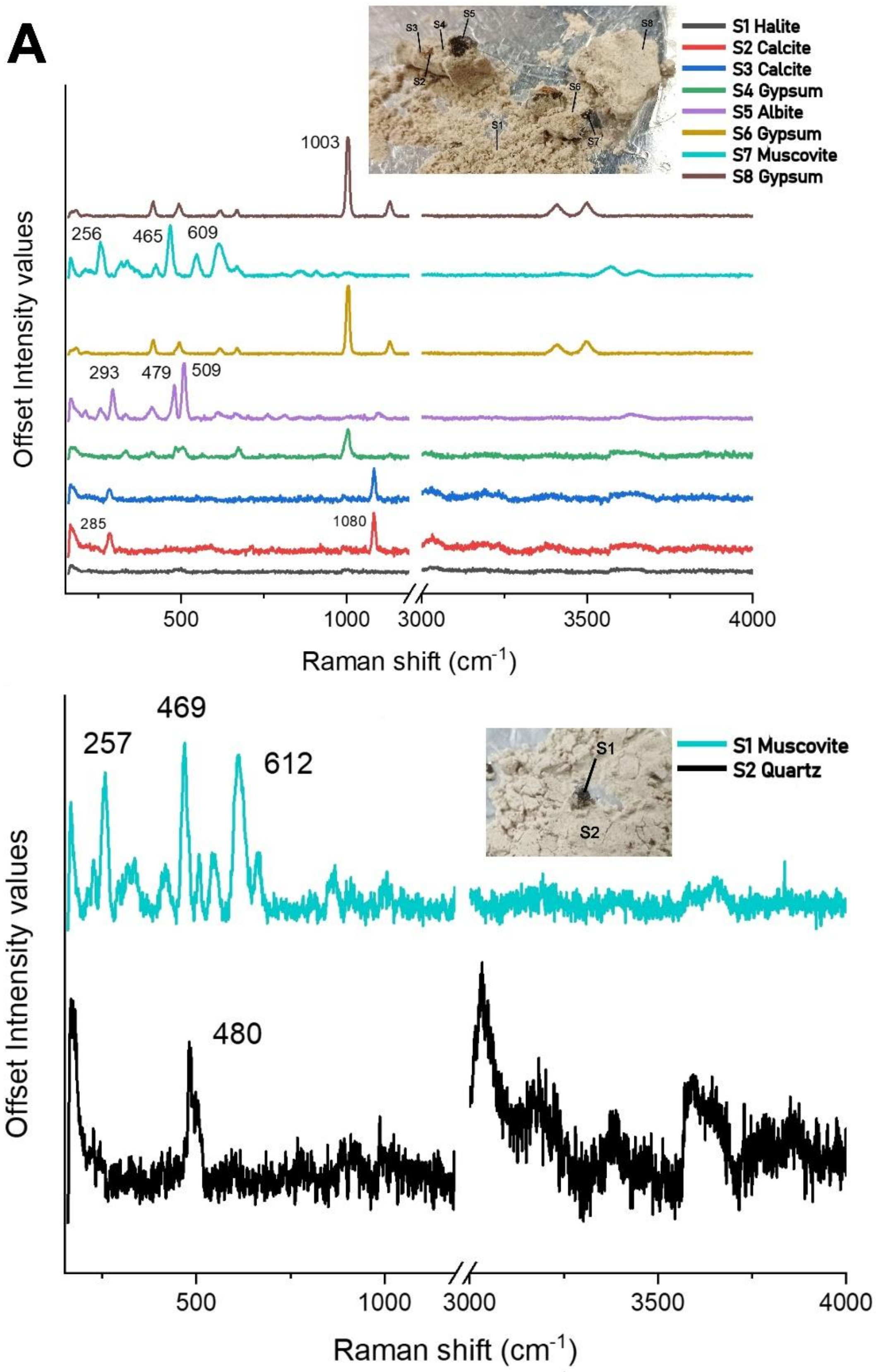

Figure S3.- Raman spectra of Mancha Blanca samples. Raman spectra were taken in different spots of the samples analyzed, where the minerals present were identified by comparison with the ADAMM database.

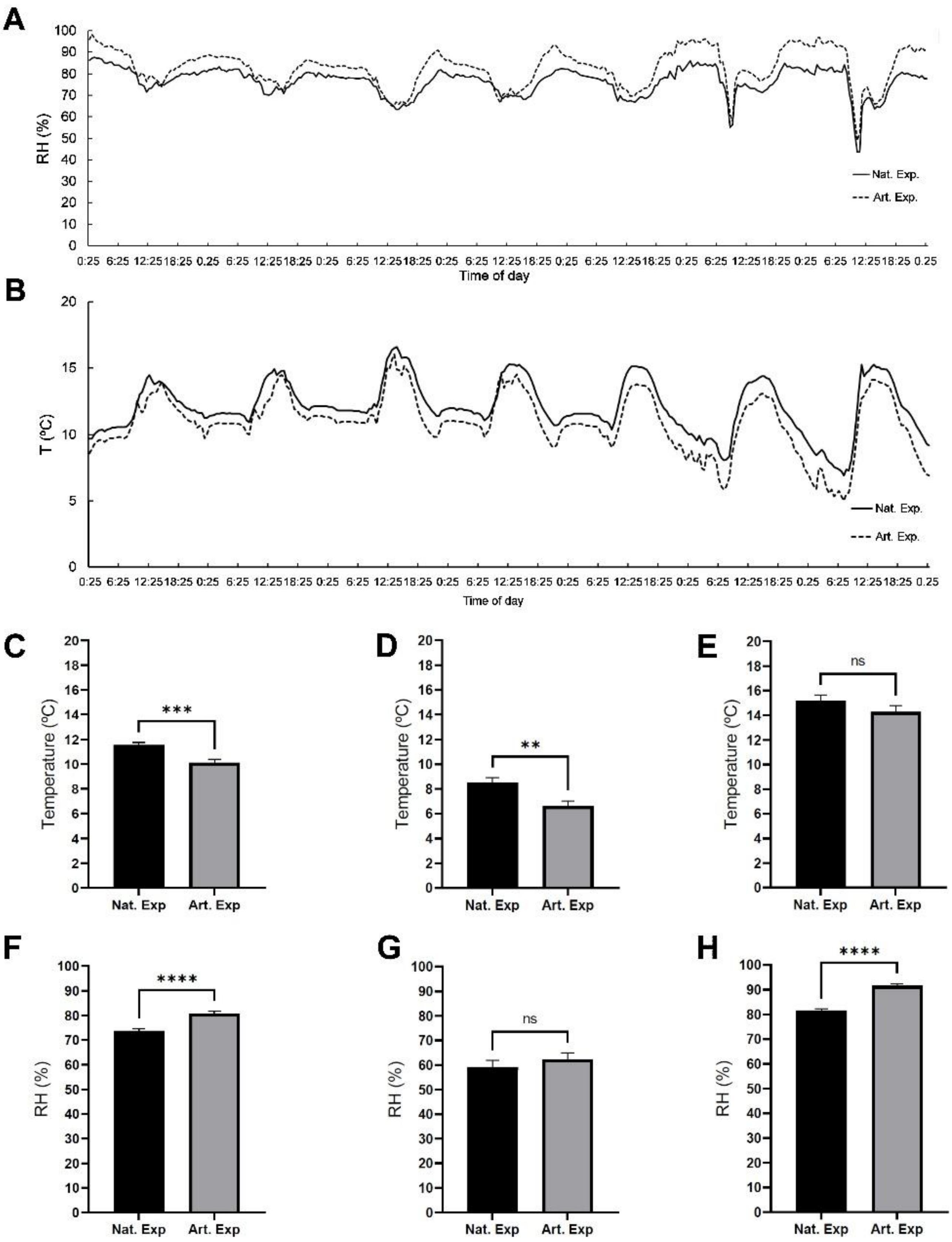

Figure S4.- Relative humidity and temperatures at Mancha Blanca site. A) Typical week variations in relative humidity, and temperatures (B). C, D and E show mean (28 days), daily minimum and daily maximum statistical differences, while F, G and H show the same variations in relative humidity between July and August of 2022. Both for RH and temperatures, p-value < 0.05, with statistical analysis performed by unpaired t-test. Error bars indicate standard error of means (n = 28).

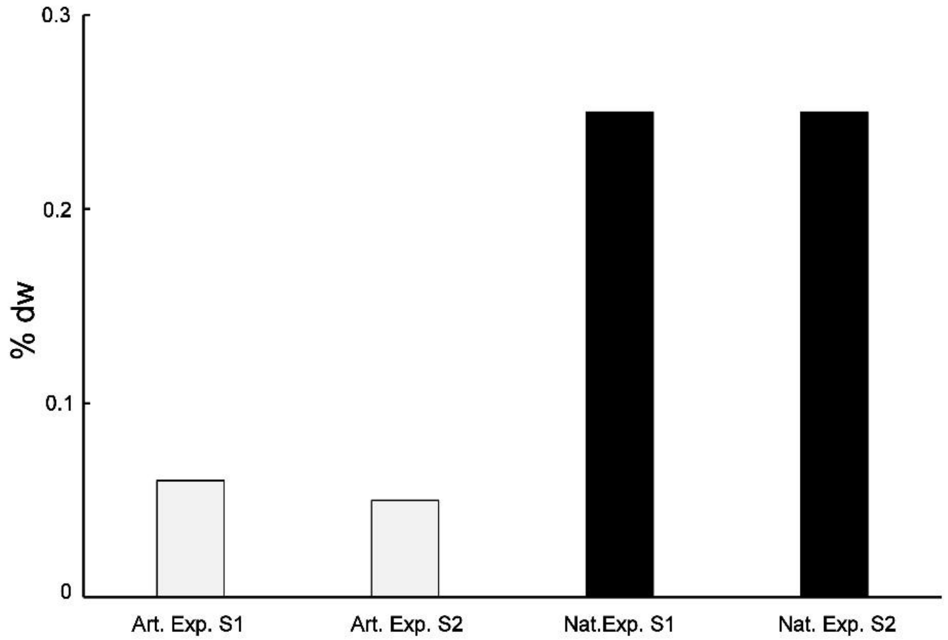

Figure S5.- Geochemical composition of the bulk organic fraction of Mancha Blanca samples. Concentration of total organic carbon (TOC) are expressed as % relative to the total dry weight (dw), measured in two randomly chosen artificially exposed (grey column) and naturally exposed samples (black column).

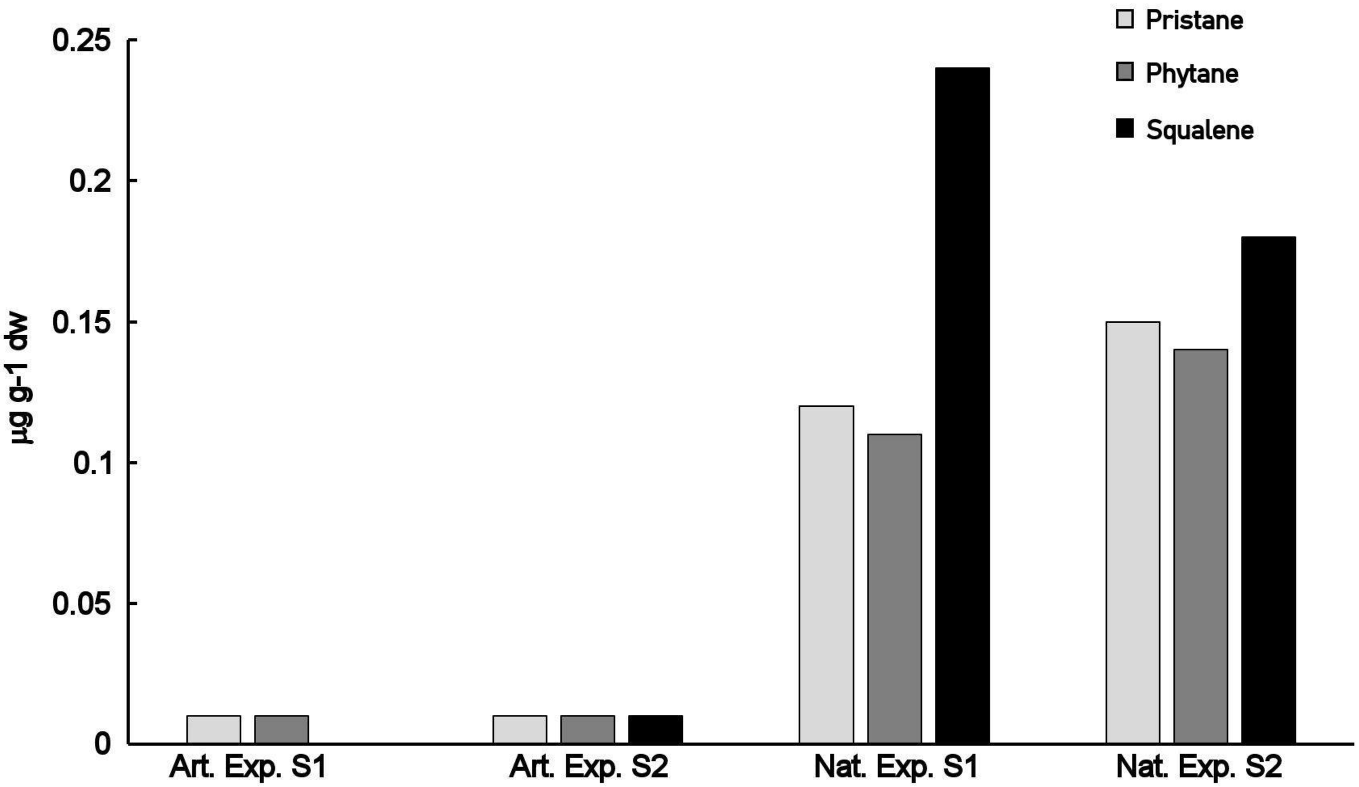

Figure S6.- Concentration (µg·g^{-1} dw) of isoprenoids (pristane, phytane and squalene) in Mancha Blanca samples. Measurements were performed in two randomly chosen artificially exposed and naturally exposed samples.

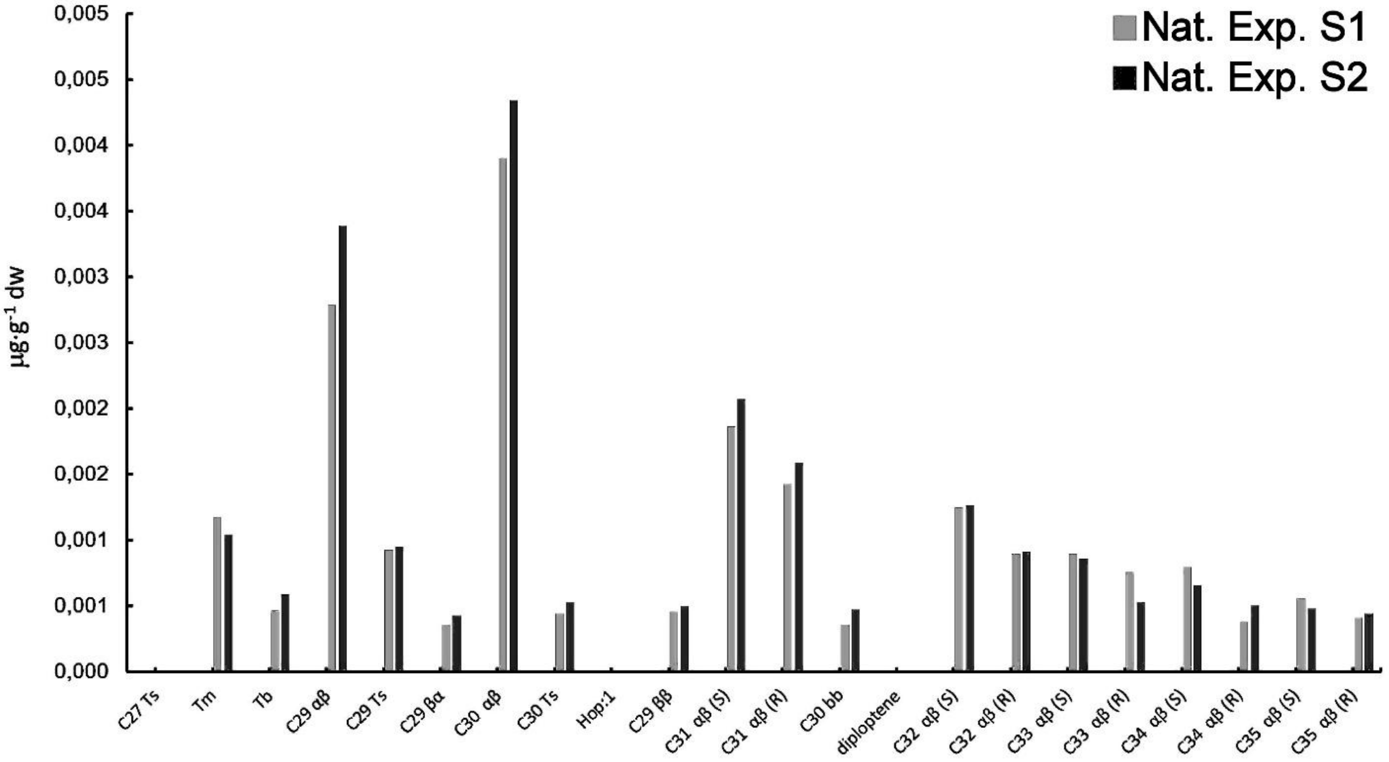

Figure S7.- Concentration (μg·g^{-1} dw) of hopanoids in naturally exposed Mancha Blanca samples. Measurements were performed in two randomly chosen artificially exposed and naturally exposed samples. Black and light gray bars correspond to the two naturally exposed samples. No hopanoids were detected in artificially exposed samples.

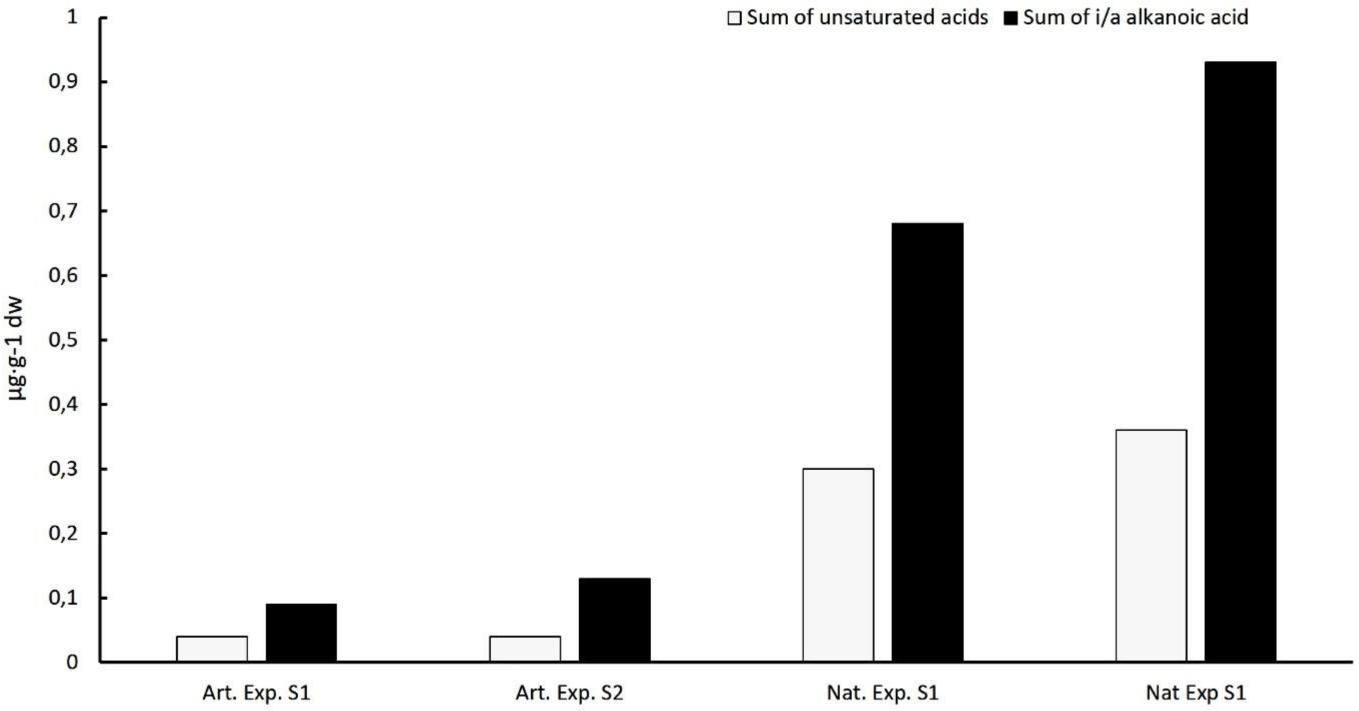

Figure S8.- Concentration (µg·g^{-1} dw) of unsaturated fatty acids (grey columns) and *iso*/*anteiso* fatty acids (black columns) in Mancha Blanca samples. Measurements were performed in two randomly chosen artificially exposed and naturally exposed samples.

Supplementary Tables 1 to 5

Table S1.- Determination of the mineralogical composition (%) of Mancha Blanca samples by X-ray fluorescence.

	SiO_2	Al_2O_3	Fe_2O_3	MnO	MgO	CaO	Na_2O	K_2O	TiO_2	P_2O_5	L.O.I	Total
Naturally Exposed	55,28	11,50	4,41	0,15	1,83	6,65	6,33	2,85	0,64	0,14	9,94	99,72
Artificially exposed	64,01	12,57	3,32	0,10	1,43	3,87	3,50	3,48	0,53	0,14	6,21	99,16

Table S2.- List of hydrocarbons, *n*-alkanes, alkenes, and monomethyl alkanes (ug·g^{-1} dw) detected in Mancha Blanca samples.

Compounds[a]	Art. Exp. S1	Art. Exp. S2	Nat. Exp. S1	Nat Exp S1
12	n.d.	n.d.	0.01	n.d.
13	0.001	n.d.	0.03	0.01
14	0.002	n.d.	0.05	0.05
15	0.003	n.d.	0.07	0.09
C16:1	n.d.	n.d.	0.05	0.05
16	0.004	0.01	0.12	0.13
17	0.006	0.01	0.16	0.21
Pristane	0.001	0.01	0.12	0.15
18	0.005	0.01	0.12	0.15
Phytane	0.001	0.01	0.11	0.14
2-methyl-18	n.d.	n.d.	0.05	0.06
3-methyl-18	n.d.	n.d.	0.03	0.03
19	0.009	0.01	0.24	0.30
2-methyl-19	n.d.	n.d.	0.03	0.03
3-methyl-19	n.d.	n.d.	0.02	0.03
20	0.009	0.02	0.26	0.34
2-methyl-20	n.d.	n.d.	0.03	0.04
3-methyl-20	n.d.	n.d.	0.03	0.05
21	0.011	0.02	0.29	0.41
22	0.012	0.02	0.31	0.42
23	0.012	0.02	0.28	0.40
24	0.011	0.02	0.31	0.40
25	0.011	0.02	0.25	0.35
26	0.008	0.01	0.30	0.29
27	0.006	0.01	0.18	0.22
28	0.004	0.01	0.12	0.15
Squalene	n.d.	0.01	0.24	0.18
29	0.003	0.01	0.12	0.12
30	0.002	n.d.	0.06	0.05
31	0.001	n.d.	0.05	0.03
32	n.d.	n.d.	n.d.	n.d.
33	n.d.	n.d.	n.d.	n.d.
34	n.d.	n.d.	n.d.	n.d.
35	n.d.	n.d.	n.d.	n.d.

n.d. means not detected

[a] Linear and saturated (*normal*) alkanes (*n*-alkanes) are named by simple numbers (N) that indicate the number of carbons in the chain; alkenes with a double bond (i.e. alkenes) are named as N:1; monomethyl alkanes are named as 2 or 3-methyl-N.

Table S3.- Organic geochemistry of the artificially and naturally exposed samples.
Stable carbon isotopic composition of organic carbon (‰ PDB), concentration of lipid biomarkers ($\mu g \cdot g^{-1}$ dw), and environmental proxies.

Geochemical feature	Art. Exp. S1	Art. Exp. S2	Nat. Exp. S1	Nat Exp S2
$\delta^{13}C$ (‰)	n.m.	n.m.	-26.6	-25.7
Sum of *n*-alkanes (C_{12}-C_{35})[a]	0.12	0.18	3.33	4.11
alkenes (C_{16})[b]	n.d.	n.d.	0.05	0.05
Sum of MMe- alkanes[c]	n.d.	0.01	0.18	0.23
Sum of *n*- alkanoic acids ($C_{11:0}$-$C_{24:0}$)[d]	1.32	1.82	5.80	9.36
Sum of *n*-alkanols (C_{12}-C_{24})[e]	0.01	0.01	0.25	0.26
Sum of unsaturated alkanols (C_{22}-C_{24})[f]	n.d.	n.d.	0.02	0.01
Sum of cholesterol and derivates[g]	n.d.	n.d.	0.07	0.04
Coprostanol	n.d.	n.d.	n.d.	n.d.
phytosterols[h]	n.d.	n.d.	0.04	0.04
ACL *n*-alkanes (C_{13}-C_{35})[i]	22	22	22	22
CPI *n*-alkanes (C_{13}-C_{35})[j]	1.08	1.09	1.03	1.09
Pr /Py	1.1	1.1	1.1	1.1
n- alkanoic acids / *n*-alkanes	10.9	9.9	1.7	2.3

[a] *n*- Sum of *normal* alkanes (i.e. linear and saturated) chains in the range of carbon units indicated in brackets.
[b] Sum of alkanes with one unsaturation (i.e. double bond).
[c] Sum of monomethylated alkanes.
[d] *n*- Sum of *normal* alkanoic acids (i.e. linear and saturated) chains in the range of carbon units indicated in brackets.
[e] *n*- Sum of *normal* alkanols (i.e. linear and saturated) chains in the range of carbon units indicated in brackets.
[f] Sum of alkanols with double bonds (1 unsaturation).
[g] Sum of cholesterol, cholestan-3-ol, (3β,5β)-, and cholest-7-en-3-ol, (3β,5α)-.
[h] Sum of β-sitosterol and stigmastanol.
[i] ACL $_{i\text{-}n}$ average chain length = Σ(i·Xi +... + n·Xn)/ ΣXi +... + Xn), where X is concentration (van Dongen et al. 2008).
[j] Proxy indicative of biomass maturity (Hedges and Prahl, 1993). CPI $_{i\text{-}n}$, carbon preference index = ½ Σ (Xi + Xi+2 +... + Xn)/ Σ(Xi-1 + Xi+1 +... + Xn-1) + 1/2Σ (Xi +Xi+2 +... + Xn)/ Σ (Xi+1 + Xi+3 +... + Xn+1), where X is concentration (van Dongen et al. 2008).
n.m. means not measured.

Table S4.- List of alkanols and sterols detected in Mancha Blanca samples (ug·g^{-1} dw).

Compounds[a]	Art. Exp. S1	Art. Exp. S2	Nat. Exp. S1	Nat Exp S2
12	n.d.	n.d.	n.d.	n.d.
14	0	0	0	0
15	n.d.	n.d.	0.01	0.01
15	n.d.	n.d.	0.01	0.01
15	n.d.	n.d.	0.05	0.08
16	n.d.	n.d.	0.01	0.01
17	n.d.	n.d.	n.d.	n.d.
18:1	n.d.	n.d.	n.d.	n.d.
18	n.d.	n.d.	0.08	0.08
19	n.d.	n.d.	0.01	n.d.
20:1	n.d.	n.d.	0.02	0.01
20	n.d.	n.d.	0.05	0.04
21	n.d.	n.d.	n.d.	n.d.
22:1	n.d.	n.d.	n.d.	n.d.
22	n.d.	n.d.	0.03	0.01
23	n.d.	n.d.	0.01	n.d.
24:1	n.d.	n.d.	n.d.	n.d.
24	n.d.	n.d.	n.d.	n.d.
Cholesterol	n.d.	n.d.	0.07	0.04
Coprostanol	n.d.	n.d.	n.d.	n.d.
β-Sitosterol	n.d.	n.d.	0.03	0.03
Stigmastanol	n.d.	n.d.	0.01	0.01
Cholestan-3-ol, (3β,5β)-	n.d.	n.d.	n.d.	n.d.
Cholest-7-en-3-ol, (3β,5α)-	n.d.	n.d.	n.d.	n.d.

n.d. means not detected.

[a] Linear and saturated (*normal*) alkanols (*n*-alkanols) are named by simple numbers (N) that indicate the number of carbons in the chain; alkanols with a double bond are named as N:1.

Table S5.- List of fatty acids detected in Mancha Blanca samples (ug·g^{-1} dw).

Compounds[a]	Art. Exp. S1	Art. Exp. S2	Nat. Exp. S1	Nat Exp S1
$C_{11:0}$	n.d.	n.d.	0.02	0.07
$C_{12:0}$	n.d.	0.01	0.04	0.06
$C_{13:0}$	n.d.	n.d.	n.d.	0.01
$iC_{14:0}$	0.01	0.02	0.09	0.10
$C_{14:0}$	0.22	0.29	0.98	1.48
$iC_{15:0}$	0.08	0.10	0.49	0.66
$aC_{15:0}$	n.d.	0.01	0.05	0.09
$C_{15:1\omega5}$	n.d.	n.d.	0.02	0.02
$C_{15:0}$	0.03	0.05	0.28	0.33
$iC_{16:0}$	n.d.	n.d.	n.d.	n.d.
$aC_{16:0}$	n.d.	n.d.	0.02	0.03
$C_{16:1\omega7}$	0.01	0.01	0.11	0.13
$C_{16:0}$	0.72	0.95	2.88	4.92
$iC_{17:0}$	n.d.	n.d.	0.01	0.02
$aC_{17:0}$	n.d.	n.d.	0.02	0.03
$C_{17:1\omega9}$	n.d.	n.d.	0.02	0.02
$C_{17:0}$	0.01	0.03	0.12	0.15
$C_{18:2\omega10,13}$	n.d.	n.d.	n.d.	n.d.
$C_{18:2\omega6,9}$	n.d.	n.d.	n.d.	n.d.
$C_{18:1\omega9}$	0.02	0.02	0.13	0.16
$C_{18:1\omega5}$	n.d.	n.d.	n.d.	n.d.
$C_{18:0}$	0.33	0.46	1.25	2.19
$C_{19:1\omega9}$	n.d.	0.01	0.02	0.02
$C_{19:0}$	n.d.	n.d.	n.d.	n.d.
$C_{20:0}$	0.01	0.01	0.10	0.06
$C_{21:0}$	n.d.	n.d.	0.01	0.01
$C_{22:0}$	n.d.	n.d.	0.05	0.02
$C_{23:0}$	n.d.	n.d.	0.01	0.01
$C_{24:0}$	n.d.	n.d.	0.06	0.04

n.d. means not detected.

[a] The alkanoic acids are named as N:n, where N indicates the total number of carbon in the chain and n the number of double bonds. In polyunsaturated acids, the positions of the double bonds are indicated in the omega notation (i.e., respect to the end carbon). *Iso* (*i*N:0) and *anteiso* (*a*N:0) alkanoic acids are alkanoic acids with a methyl group in position N-1 or N-2, respectively.
